# Tenomodulin knockout mice exhibit worse late healing outcomes with augmented trauma-induced heterotopic ossification of Achilles tendon

**DOI:** 10.1038/s41419-021-04298-z

**Published:** 2021-11-05

**Authors:** Manuel Delgado Caceres, Katharina Angerpointner, Michael Galler, Dasheng Lin, Philipp A. Michel, Christoph Brochhausen, Xin Lu, Adithi R. Varadarajan, Jens Warfsmann, Richard Stange, Volker Alt, Christian G. Pfeifer, Denitsa Docheva

**Affiliations:** 1 Experimental Trauma Surgery, Department of Trauma Surgery, University Regensburg Medical Centre, Regensburg, Germany; 2 Hand, Elbow and Plastic Surgery Department, Schön Klinik München Harlaching, Munich, Germany; 3 Department of Trauma Surgery, Caritas Hospital St. Josef, Regensburg, Germany; 4 Orthopaedic Center of People’s Liberation Army, The Affiliated Southeast Hospital of Xiamen University, Zhangzhou, China; 5 Department of Trauma-, Hand-, and Reconstructive Surgery, University Hospital Münster, Münster, Germany; 6 Institute of Pathology, University of Regensburg, Regensburg, Germany; 7 Division of Personalized Tumor Therapy, Fraunhofer Institute for Toxicology and Experimental Medicine, Regensburg, Germany; 8 Department of Regenerative Musculoskeletal Medicine, Institute for Musculoskeletal Medicine, University Hospital Münster, Westfälische Wilhelms-University, Münster, Germany; 9 Clinic and Policlinic for Trauma Surgery, University Regensburg Medical Centre, Regensburg, Germany; 10 Department of Musculoskeletal Tissue Regeneration, Orthopaedic Hospital König-Ludwig-Haus, University of Würzburg, Würzburg, Germany

**Keywords:** Biological sciences, Cell biology

## Abstract

Heterotopic ossification (HO) represents a common problem after tendon injury with no effective treatment yet being developed. Tenomodulin (*Tnmd*), the best-known mature marker for tendon lineage cells, has important effects in tendon tissue aging and function. We have reported that loss of *Tnmd* leads to inferior early tendon repair characterized by fibrovascular scaring and therefore hypothesized that its lack will persistently cause deficient repair during later stages. *Tnmd* knockout (*Tnmd*^*−/−*^) and wild-type (WT) animals were subjected to complete Achilles tendon surgical transection followed by end-to-end suture. Lineage tracing revealed a reduction in tendon-lineage cells marked by ScleraxisGFP, but an increase in alpha smooth muscle actin myofibroblasts in *Tnmd*^−^^*/−*^ tendon scars. At the proliferative stage, more pro-inflammatory M1 macrophages and larger collagen II cartilaginous template were detected in this group. At the remodeling stage, histological scoring revealed lower repair quality in the injured *Tnmd*^*−/−*^ tendons, which was coupled with higher HO quantified by micro-CT. Tendon biomechanical properties were compromised in both groups upon injury, however we identified an abnormal stiffening of non-injured *Tnmd*^*−/*^^−^ tendons, which possessed higher static and dynamic E-moduli. Pathologically thicker and abnormally shaped collagen fibrils were observed by TEM in *Tnmd*^*−/−*^ tendons and this, together with augmented HO, resulted in diminished running capacity of *Tnmd*^*−/−*^ mice. These novel findings demonstrate that *Tnmd* plays a protecting role against trauma-induced endochondral HO and can inspire the generation of novel therapeutics to accelerate repair.

## Introduction

Heterotopic ossification (HO) is the process of ectopic cartilage formation followed by endochondral ossification of soft tissues such as tendons [1, 2]. Following open Achilles tendon reconstructions, up to 28% of patients suffer from HO [3, 4]. Trauma-induced HO significantly impacts tendon viscoelastic properties [5, 6], leading to serious decrease in patients quality of life. To date, no satisfactory therapeutic targets have been developed. The natural healing process of tendons, divided in early (inflammatory) and late (proliferative and remodeling) stages, is known to be inefficient, long-lasting and influenced by anatomic location and local mechanical environment [7]. Dysregulated inflammatory response in tendon lesions drives persistent fibrosis, misguided stem/progenitor cell differentiation and leads to HO [8, 9]. The discrete molecular mechanisms and origin, existence and functions of different cell types throughout the long repair process are still not fully understood. Tendon-resident cells are marked by the expression of Scleraxis (*Scx*) [10], and their lineage tracking by using Scleraxis-GFP (*ScxGFP)* reporter mice [11] has been crucial for the identification of cells triggering intrinsic tissue repair [12–15]. On the other hand, extrinsic epitenon/paratenon cells, expressing alpha smooth muscle actin (αSMA), and persistent pro- inflammatory M1 macrophages, have been shown to initiate pathologic scarring, chronic low-grade inflammation, poor tissue remodeling and HO [12, 15, 16].

Tenomodulin *(Tnmd)* gene, predominantly expressed in tendons and ligaments, encodes a type II transmembrane glycoprotein with a cleavable C-terminal cysteine-rich domain, that is subsequently deposited in the extracellular matrix (ECM) proximal to type I collagen fibrils [17–19]. The C-terminal hydrophobic tail exerts a dual role stimulating proliferation of tendon cells [20], whilst inhibiting vascular cell migration [21]. It has also been demonstrated that *Tnmd* regulates the proliferation, senescence and differentiation of tendon stem/progenitor cells (TSPC) [22]. Moreover, we have previously shown that TSPCs derived from *Tnmd*-knockout mice exhibit defective in vitro collagen remodeling [23]. Further, a significant shift towards thicker and stiffer collagen fibrils was identified within non-injured mutant Achilles tendons [20, 24], resulting in profoundly hampered performance in forced endurance running tests [24]. Next, when subjected to surgically induced full thickness Achilles tendon rupture [25, 26], *Tnmd*^−/−^ mice displayed inferior scar tissue characterized by significantly reduced cell density and proliferation, increased vessel infiltration, and accumulation of M1 macrophages, adipocytes and erroneous ECM after 8 days [26]. Here, we implemented the established surgical model and carried out a longitudinal investigation up to day 100 with *Tnmd*^*−/−*^ and wild type (WT) animals, by combining lineage tracking, immuno-histomorphometry, transmission electron microscopy (TEM), micro-CT (µCT), viscoelastic biomechanical testing, voluntarily running recording, and single cell RNA-sequencing to diagnose for the first time transcriptional differences between both genotypes. Based on our previous findings we hypothesized that the lack of *Tnmd* will persistently cause deficient repair during the later proliferative and remodeling stages, and will lead to a significant endochondral heterotopic ossification of Achilles tendons.

## Material and methods

### Study approval

All procedures regarding animal handling, husbandry, surgical model, pre-, and postoperative animal care were approved by the Animal Care and Use Committee of the Lower Franconia Government, Wuerzburg, Germany (Grant No. 55.2-2532-2-466). In the group size calculations, for the type one error and power, the standard values α = 0.05 and 1–β = 0.8 were used, respectively.

### Animal model and surgical procedure

Constitutive *Tnmd* knockout (*Tnmd*^*−/−*^) mice and WT littermates, as well as *ScxGFP* mice were previously described by Docheva et al. [20] and Pryce et al. [11], respectively. Conditional Tnmd flox/flox strain is not yet available. *Tnmd* gene is located on the X-Chromosome, therefore the WT group comprised hemizygous male, homozygous-, and heterozygous female mice. All analyzed animals were maintained on the C57BL/6 J background. The experimental design and the group distribution including n-numbers are shown in Supplementary Fig. 1A. Skeletally mature animals (6-month old) were operated according to Lin et al. [26]. Detailed description of the surgical procedure provided in Supplementary Information and Supplementary Fig. 1B, C.

### Immunohistochemistry and histomorphometry

Mouse hindlimbs (exact n-numbers shown as dot plot as well as given in figure legends) were fixed overnight in fresh 4% paraformaldehyde (PFA, Merck, Darmstadt, Germany) in phosphate-buffered saline (PBS; pH 7.4) or in 95% ethanol- 5% glacial acetic acid. Specimens were decalcified in 10% ethylen diamine tetraacetic acid (EDTA)/phosphate buffered saline (PBS) pH 8.0 (Sigma-Aldrich, Munich, Germany) for 4 weeks, embedded in cryoprotective media and sectioned at 10 µm, every 10^th^ slide was stained with Hematoxylin-Eosin following standard protocol. Tissue sections with equivalent regional planes between genotypes were selected for detailed investigation. Histological scoring was carried out by two independent observers at day 21 (*n* = 10/genotype) and 100 (*n* = 11/genotype) according to Stoll et al. [27] (Supplementary Table 1). Detailed description of immunohistochemistry, immunofluorescence stainings and automated quantitative image analysis provided in Supplementary information.

### Transmission electron microscopy (TEM)

Injured and contralateral Achilles tendons were explanted at day 21 and 100 post-injury (*n* = 3 genotype/time point), fixed in Karnovsky (0.1 M cacodylate-buffer with 2.5% glutaraldehyde and 2% paraformaldehyde). Ultrathin sections (0.08 μm) were analyzed in a LEO912AB transmission electron microscope (Zeiss, Oberkochen, Germany) operating at 100 kV. Findings were documented with a side-mounted 2 k x 2 k-CCD-camera (TRS, Moorenweis, Germany). The iTEM software (Olympus, Tokyo, Japan) was used to measure collagen fibril diameter. Five images (40000x magnification)/tendon (*n* = 2/group/time point) were used for quantification, resulting in average of 2800 fibrils/group/time point being analyzed (Supplementary Table 2).

### Micro-computed tomography (µCT)

Muscle-Achilles tendon-calcaneus complex (contralateral, non-injured control, and injured Achilles tendons) were excised and collected 100 days post-injury (*n* = 7–8 animals/genotype), fixed as described above and scans were performed with the µCT system Phoenix v|tome|x s 240/180 (GE Sensing & Inspection Technologies, Frankfurt am Main, Germany). The scanning parameters were as follows: 50 kV voltage, 620 μA current, 500 ms time, 2000 images, voxel size 10 μm. The 3D images, the surface area and volume parameters were obtained with the software Volume Graphics VG Studio Max 2.2.3 (Volume Graphics, Heidelberg, Germany). Detailed description provided in Supplementary information.

### Biomechanical testing

Mouse hindlimbs were explanted, wrapped in PBS-soaked gauze and stored at −20 °C until testing. On the testing day, hindlimbs were thawed for 30 min at RT. The viscoelastic biomechanical tests were performed with day 100 specimens (non-injured, contralateral and injured, *n* = 8–14/genotype). Biomechanical testing at day 21 was not carried out, because the scar tissue is dominated by cells and literature has previously reported that viscoelastic properties of rodent Achilles tendons are greatly compromised in first weeks after injury [28, 29]. A LM1 machine (TA Instruments, New Castle, USA) and custom-made clamps, securing the calcaneal and myotendinous junction ends, were used. All tests were performed in PBS-bath at RT. The testing protocol was based on Dourte et al. [30] and Hochstrat et al. [31]. Detailed description provided in Supplementary information. The analysis of the data was carried out with a custom-written Matlab software (MathWorks, Natick, Massachusetts, USA) protocol.

### Voluntary running tests

Mice (*n* = 7–10/genotype) were acclimatized to the experimental cage, containing a standard free-spinning mouse running wheel (12 cm diameter) equipped with wired bike computer BC 5.16 (Sigma Sport, Neustadt, Germany) for 3 days. At day 4, mice were placed individually in the experimental cage, left overnight (12 h) to voluntarily use the wheel and the running distance was recorded.

### Single cell RNA-Sequencing (scRNA-Seq)

Control, non-injured *Tnmd*^*−/−*^ *ScxGFP*^+^ and WT *ScxGFP*^*+*^ Achilles tendons (*n* = 2) were explanted and GFP^*+*^ cells were isolated by collagenase digestion (8 h) and filtration according to [22]. Next, cells (*n* = 20/genotype) were resuspended in PBS, placed on Adcell diagnostic slides (Thermo Fisher, Waltham, Massachusetts, USA), picked up in 1 µl PBS each using a micromanipulator (Patchman NP2) with pump (CellTram, both Eppendorf, Hamburg, Germany) and subsequently stored in Smart-Seq2 lysis buffer at −80 °C. The whole transcriptome amplification (WTA) and Illumina Nextera XT library preparation (Illumina, San Diego, California, USA) were performed as described by Picelli et al. [32]. The libraries were quantified using the KAPA Library Quantification kit (Roche Diagnostics, Mannheim, Germany), pooled in equimolar amounts and sequenced paired-end with read length of 2 × 150 bp and yield of 30 million reads per library on an Illumina HiSeq. In total, six *ScxGFP*^+^ cells (*n* = 6/genotype) were subjected to scRNA-Seq. Bioinformatic analysis is described in Supplementary information.

### Statistical analysis

Statistical calculations and graphs preparation were performed with GraphPad Prism 7 (San Diego, CA, USA). The results are presented as box plots (including dot plots where each dot represents an animal) with median and interquartile range (IQR), the whiskers show minimum and maximum values. After normality Shapiro-Wilk check, two-group analyses were performed, if not otherwise stated, with 2-tailed parametric unpaired Student’s *t*-test. Multi-group comparisons were evaluated by one-way ANOVA with Bonferroni post-hoc test. Differences were considered statistically significant according to p-values of *p < 0.05, **p < 0.01, ***p < 0.001 and ****p < 0.0001.

## Results

### *Tnmd*^*−/−*^ scars harbor significantly higher number of αSMA^+^ cells and lower number of tendon-specific ScxGFP^+^ cells during early and late tendon healing

**Fig. 1:**
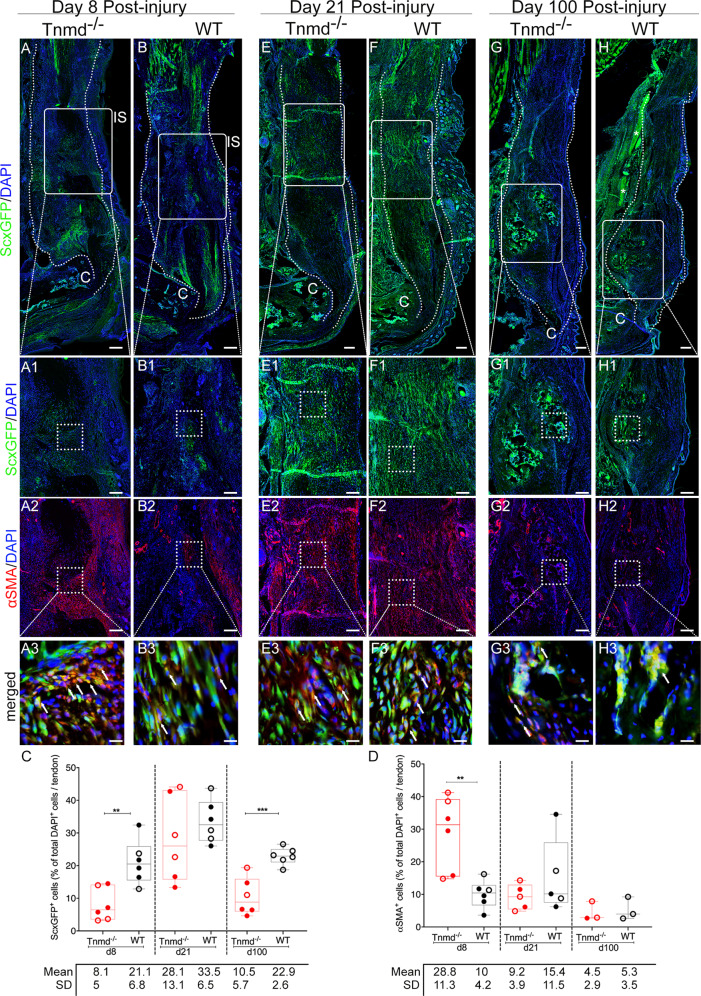
Incidence of ScxGFP^+^ and αSMA^+^ cell populations during tendon healing at day 8, 21 and 100 post-injury. Representative images of injured *Tnmd*^*−/−*^ ScxGFP and WT ScxGFP Achilles tendons at **A**, **B** day 8, **E**, **F** day 21 and **G**, **H** day 100 post-injury. A1–H1 ScxGFP^+^ cell occurrence over time. A2–H2 Corresponding immunofluorescence images of αSMA and merged images (A3–H3). White dotted lines frame Achilles tendons; white asterisks mark nerves; white arrows mark double positive ScxGFP^+^/αSMA^+^ cells. C = calcaneus, IS = injury site. Scale bar: 200 µm (panels A-F); 100 µm (panels A1–H2); 20 µm (A3–F3). Quantitative analysis of ScxGFP^+^ (**C**) and αSMA^+^ (**D**) cells in percentage of total number of DAPI^+^ cells within Achilles tendon scars. ScxGFP imaging, αSMA staining and quantification were performed with *n* = 3–6 animals/genotype/time point; each animal represented by three tissue sections. Box plots show median ± interquartile range (IQR), statistical significance was assessed with 2-tailed unpaired parametric Student’s *t*-test, *p < 0.05, **p < 0.01. Empty dot represents female mouse; filled dot represents male mouse.

Since ScxGFP^+^ and αSMA^+^ cells have been implicated as crucial players in the tendon repair process, we first investigated their abundance by lineage tracing at day 8, 21 and 100 post-injury. At the earliest time point ScxGFP^+^ cells were very scarce at the injury site (Fig. 1A–B1, C) and significantly less in the *Tnmd*^*−/−*^ group. In contrast, approx. one third of DAPI^+^ cells were positively stained for αSMA in *Tnmd*^*−/−*^ scars (Fig. 1A2, B2, D) compared to approx. one tenth in the WT (Fig. 1D). Of note, in the three examined time points single cells were ScxGFP^+^ and αSMA^+^ (Fig. 1A3–H3). At day 21, ScxGFP^+^ cell numbers were strongly increased and bridged the defect between the sutured Achilles tendons ends (Fig. 1E–F1). Furthermore, a clear decline in αSMA^+^ cells was detected in the *Tnmd*^*−/−*^ group (Fig. 1D, E2, F2). At day 100, ScxGFP^+^ and αSMA^+^ cells were present in the scar regions that appeared to be ossified (Fig. 1G–H2) and the percentage of ScxGFP^+^ cells was significantly lower in the *Tnmd*^*−/−*^ tendons compared to WT (Fig. 1C), whilst the very low abundant αSMA^+^ cells were comparable between the groups (Fig. 1D). Altogether, these data demonstrate that loss of *Tnmd* results in myofibroblast-enriched early scar tissue and significantly lower content of tendon lineage ScxGFP^+^ cells during early and late repair stages.

### At day 21 post-injury, *Tnmd*^*−/−*^ scars are characterized by larger chondrogenic template and higher number of pro-inflammatory macrophages, but lower BrdU^+^ cell count

**Fig. 2:**
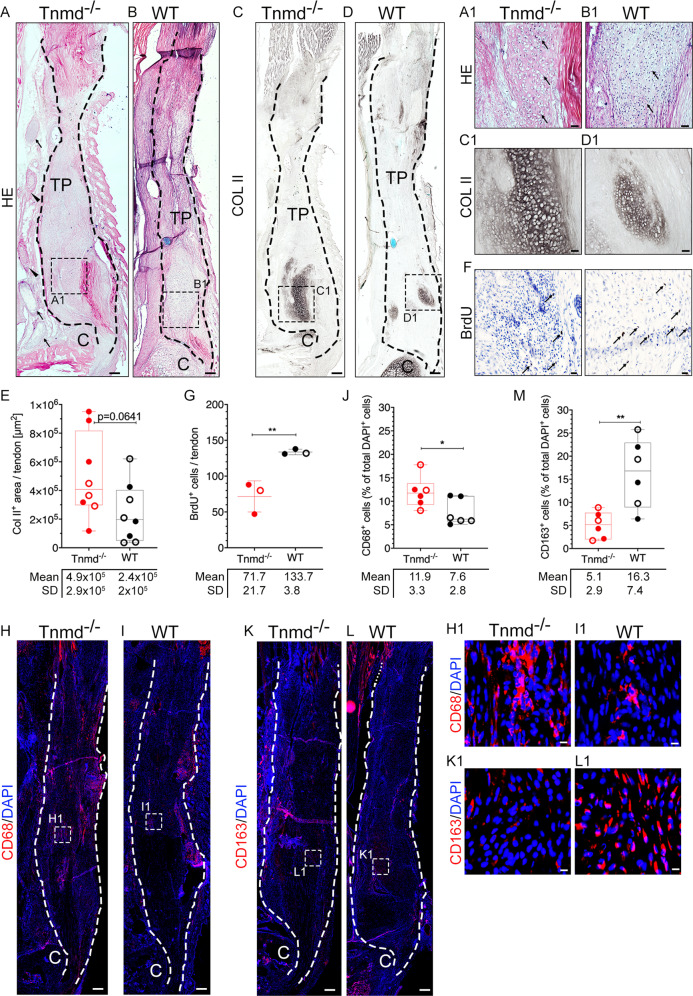
Comparison of tendon repair between *Tnmd*^*−/−*^ and WT mice at day 21 post-injury. **A**, **B** Representative mosaic HE and (A1, B1) higher magnification images. **C**, **D** Representative images and (C1, D1) higher magnification DAB images for type II collagen (COLII). **E** Quantification of COLII^+^ area/tendon. **F** BrdU staining and **G** quantification of BrdU^+^ cells/tendon. **H**, **I** Representative fluorescence images for CD68, (H1, I1) higher magnification images and **J** quantification of CD68^+^ cells. **K**, **L** Representative immunofluorescence staining for CD163, (K1, L1) higher magnification images and **M** quantification of CD163^+^ cells. Black and white dotted lines frame Achilles tendons; vessels and nerves in the peritendinous space are marked with black arrowheads and arrows, respectively; blue bright spot in **B** and **D**: cerclage 6-0 prolene suture material; black arrows in (F) mark BrdU^+^ cells. C = calcaneus, TP = tendon proper. Scale bar: 200 µm (panels A–L); 50 µm (panels A1–D1); 20 µm (panels F, H1–L1). Stainings and quantifications were performed with *n* = 3–8 animals/genotype; each animal represented by three mosaic tissue sections. Box plots show median ± IQR; statistical significance was assessed with 2-tailed unpaired parametric Student’s *t*-test, *p < 0.05, **p < 0.01. Empty dot represents female mouse; filled dot represents male mouse.

At day 21, haematoxylin-eosin (HE) staining revealed the presence of a cartilage-like nodule adjacent to the enthesis, large blood vessels and nerve fibers running in the peritendinous space of the injured tendons (Fig. 2A, B). High magnification images showed chondrocytic cells (Fig. 2A1, B1), which was confirmed by type II collagen (COLII) deposition in the territorial ECM (Fig. 2C–D1). Quantitative analysis of the COLII^+^ areas revealed a tendency of larger cartilage template in the *Tnmd*^*−/−*^ group (Fig. 2E). Nerves and blood vessels were validated and quantified by neurofilament protein-heavy chain (NEFH) and collagen type IV (COLIV) stainings, but no genotype differences were detected (Supplementary Fig. 2A–F). Histological scoring [27] (Supplementary Table 1) suggested a tendency of inferior total scores for mutant compared to WT tendons (Supplementary Fig. 2G), which was in line with broader collagen III (COLIII)-rich regions, indicative of immature ECM in the *Tnmd*^*−/−*^ tendons monitored by Herovici staining (Supplementary Fig. 3A–B1). Collagen fiber arrangement analysis in the scar tissue revealed no apparent differences between *Tnmd*^*−/−*^ and WT (Supplementary Fig. 3C–D1). Next, we evaluated the incidence of pericyte, multipotent progenitors by immunofluorescent staining for CD146, but no significant difference in the number of CD146^+^ cells was detected between the genotypes (Supplementary Fig. 2H–J). Similarly to day 8 [26], at day 21 *Tnmd*^−^^*/−*^ scars contained significantly less BrdU^+^ cells (Fig. 2F, G), higher numbers of CD68^+^ macrophages (Fig. 2H–J) and less anti-inflammatory CD163^+^ macrophages (Fig. 2K–M). Lastly, the expression of 14 tendon-related gene markers (Supplementary Fig. 3E) was evaluated by qRT-PCR. The analysis revealed the significant downregulation of 9 of the genes, besides of *Scx*, in the *Tnmd*^*−/−*^ group, which might be indicative of delayed activation of tenogenesis in the scar (Supplementary Fig. 3F). In sum, the above data reveals that certain parameters were comparable between genotypes, but the absence of *Tnmd* in the proliferative stage of the tendon healing process leads to abnormal, rather pro-inflammatory macrophage profile and reduced cell proliferation suggesting overall soft tissue destruction favouring ectopic chondrogenesis.

### The ECM of *Tnmd*^*−/−*^ tendons is distinguished by lower collagen fibril density concomitant with significantly thicker and atypically shaped collagen fibrils

**Fig. 3:**
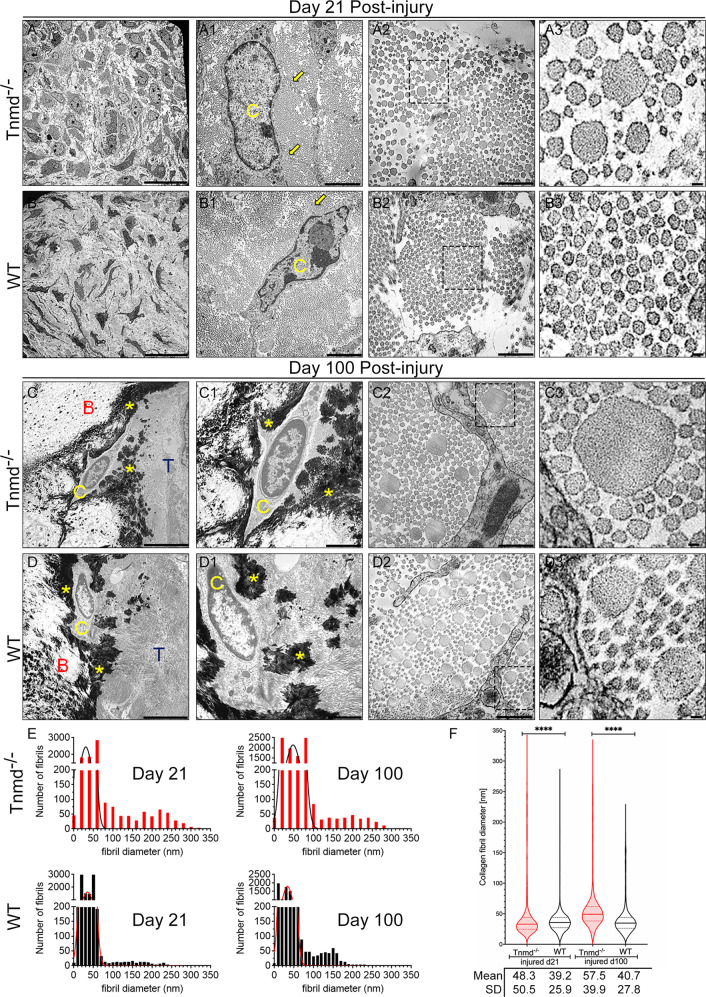
Transmission electron microscopy and collagen fibril diameter analysis of Achilles tendons at day 21 and 100 post-injury. **A**–**D** Representative 2000x, A1–D1 10000x and A2–D3 40,000x magnification images of injured *Tnmd*^*−/−*^ and WT Achilles tendons at day 21 and 100 post-injury. C = cell; B = bone; T = tendon; yellow arrow = cell protrusions; yellow asterisk = mineralized zones. Scale bars: 20 µm (panels **A**, **B**); 5 µm (panels **C**, **D**); 2 µm (A1–D1), 500 nm (panels A2–D3). **E** Histograms showing incidence of collagen fibril sizes. **F** Violin plot for collagen fibril diameter distribution, median ± IQR, statistical significance was calculated by 2-tailed unpaired nonparametric Mann–Whitney test, *p < 0.05, **p < 0.01, ***p < 0.001, ****p < 0.0001. Day 21, *n* = 2 animals/genotype; day 100, *n* = 2 animals/genotype.

Tendon ECM ultrastructure was investigated by transmission electron microscopy (TEM) and quantitative analyses of collagen fibril diameter distribution. At day 21, representative TEM images of injured *Tnmd*^*−/−*^ and WT Achilles tendons revealed hypercellularity, decreased fibril size and density, and broader gaps between cells and fibrils (Fig. 3A–B3) in comparison to the non-injured controls (Supplementary Fig. 4A–D1). Quantitative analyses showed that in the WT group the majority of the fibrils were clustered in the range of 10–50 nm diameters. In contrast, scattered and thicker collagen fibrils, reaching diameters of up to 340 nm, with irregular rough outlines were significantly enriched in the *Tnmd*^*−/−*^ tendons (Fig. 3A3, B3, E and F). This pattern of collagen fibril shift towards significantly large sizes in *Tnmd*^*−/−*^ was consistent in the injured setting at day 100 (Fig. 3C–D3, E, and F), as well as in the *Tnmd*^*−/−*^ contralateral tendons from day 21 (6 month-old) and 100 (9 month-old) (Supplementary Fig. 4A2–D2, E). In the late post-injury stage mineral deposits within the ECM and osteocyte-like cells, a sign of HO, were detected in both, *Tnmd*^*−/−*^ and WT injured tendons (Fig. 3C–D1). Taken together, the TEM imaging reproducibly demonstrated a *Tnmd*^*–/–*^-specific phenomenon, namely significant fibril thickening in both conditions, before and after injury, underpinning that *Tnmd* acts as a regulatory factor of collagen fibrillogenesis.

### Loss of *Tnmd* leads to significantly higher trauma-induced heterotopic ossification of Achilles tendon

**Fig. 4:**
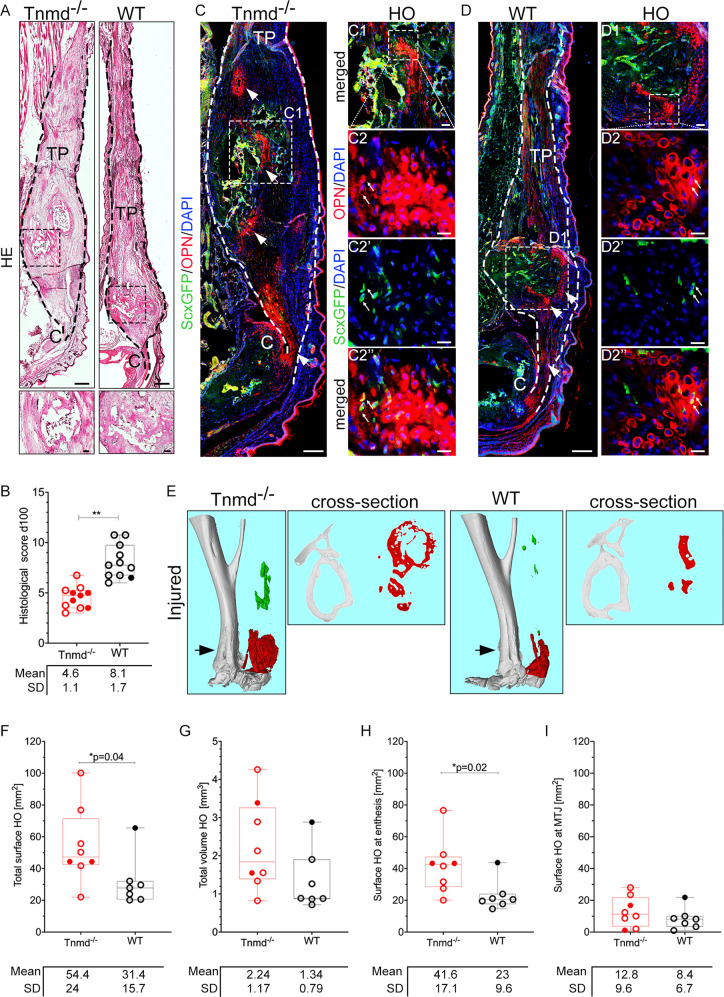
Comparison of tendon repair between *Tnmd*^*−/−*^ and WT mice at day 100 post-injury. **A** Representative HE mosaic images (bottom panel, higher magnification). **B** Histological scoring at 100 days post-injury. **C**, **D** Representative fluorescent images and C1–D2” higher magnification images of Osteopontin (OPN)-staining of *Tnmd*^*−/−*^ *ScxGFP* and WT *ScxGFP* tendons. Black and white dotted lines frame Achilles tendons; white arrows in **C**, **D** mark OPN^+^-stained regions. White arrows in C2–D2” mark double positive *ScxGFP*^+^/ OPN^+^ cells. C = calcaneus, TP = tendon proper. Scale bar: 200 µm (**A** panel); 100 µm, (**C**, **D** panels); 50 µm (C1, D1 panels); 20 µm (C2–D2” panels). **E** Representative longitudinal and cross-sectional µCT scans at day 100 post-injury. Quantification of total HO surface and volume (**F**, **G**), HO surface at the enthesis (**H**), and MTJ (**I**) in Achilles tendons 100 days post-injury. Histological scoring and µCT was performed with *n* = 7–11 animals/genotype. OPN staining was performed with *n* = 3 animals/genotype, each animal represented by three tissue sections. Box plots show median ± IQR; statistical was evaluated by 2-tailed unpaired parametric Student’s *t*-test for histological scoring, and unpaired non-parametric Mann–Whitney U test for µCT analysis, *p < 0.05, **p < 0.01. Empty dot represents female mouse; filled dot represent male mouse.

At the late repair stage (day 100), HE staining revealed that the cartilage template was replaced with prevalent bone-like tissue located proximally of the entheses (Fig. 4A). Moreover, histological scoring [27] (Supplementary Table 1) showed significantly inferior values for the mutant group (Fig. 4B). The most remarkable differences were regarding larger bone nodules containing trabecular space, poorer cell alignment in the fibrous regions of the tendon and looser ECM judged by faint Eosin staining. To exclude dystrophic calcification we performed immunofluorescence staining with the osteogenic marker osteopontin (OPN) (Fig. 4C–D1) which demonstrated broader OPN^+^ domain and higher protein levels in the *Tnmd*^*−/−*^ group. In the bone nodules, sparse double positive ScxGFP^+^/OPN^+^ cells were visible (Fig. 4C2–D2”). Next, to precisely quantify if the HO was higher in the injured *Tnmd*^*−/−*^ tendons we employed µCT scanning and quantification. First, scans of contralateral and non-injured, control tendons showed in the 9-month old animals age-related occurrence of a scarce in-tendinous HO, which was comparable between the genotypes (Supplementary Fig. 5A–D). With regards to the injured setting, longitudinal and cross-sectional representative images demonstrated a bipolar HO distribution in the tendon with the enthesial-adjacent site pervading the myotendinous junction (MTJ)-adjacent site (Fig. 4E). Total HO surface in the injured *Tnmd*^*−/−*^ tendons was significantly higher than the WTs (Fig. 4F) and similar tendency was observed for total HO volume (Fig. 4G). The measured HO surface at the enthesis was significantly increased in *Tnmd*^*−/−*^ injured tendons compared to WT (Fig. 4H), while the MTJ-adjacent HO surface was comparable between the genotypes (Fig. 4I). In order to disregard that *Tnmd*-deficiency affects bone quality, regional calcaneal and tibiofibular bone volume and surface of specimens from the same groups were quantified and no significant differences were detected (Supplementary Fig. 5E–I). Lastly, qRT-PCR analysis revealed a significantly higher mRNA levels of the majority of assessed genes in *Tnmd*^*−/−*^ tendons (Supplementary Fig. 3G), which might be indicative of still ongoing tenogenesis. Hence, these results report for the first time that lack of *Tnmd* causes overall worsening of the late stage of tendon repair process coupled with significantly augmented trauma-induced HO.

### *Tnmd*^*−/−*^ tendons are naturally stiffer and display significantly increased static and dynamic Elastic moduli; post-injury, tendon viscoelastic properties are profoundly weakened in both genotypes

**Fig. 5:**
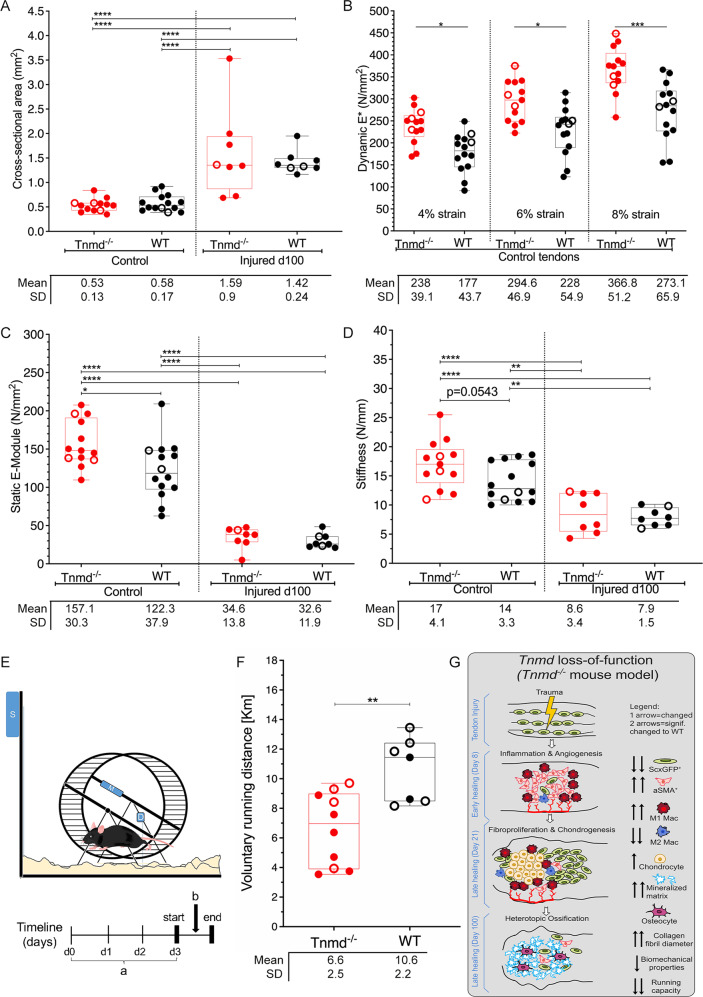
Viscoelastic biomechanical testing of non-injured, control and injured Achilles tendons, and voluntary running tests at day 100 post-injury. **A** Cross-sectional area of non-injured, control and injured Achilles tendons. **B** Dynamic Elastic modulus in control Achilles tendons at 4%, 6% and 8% strain. **C** Static Elastic modulus and **D** stiffness of non-injured, control and injured Achilles tendons. Biomechanical testing was performed with *n* = 8–14 animals/genotype. Box plots show median ± IQR; statistical analysis was performed with one-way ANOVA and Bonferroni post-hoc test. **E** Test setup (upper drawing) and timeline (bottom drawing) for voluntary running tests. **F** Recorded running distance; tests were performed with *n* = 7–10 animals/genotype. Box plots show median ± IQR; statistical significance assessed with 2-tailed unpaired parametric Student’s *t*-test, *p < 0.05, **p < 0.01, ***p < 0.001, ****p < 0.0001. Empty dots represent female mouse; filled dots represent male mouse. **G** Graphical synopsis of the major phenotypic changes in *Tnmd*^*−/−*^ animals over the time course after injury.

We subjected non-injured, control and injured *Tnmd*^*−/−*^ and WT Achilles tendons to a viscoelastic biomechanical testing at day 100 post-injury (Supplementary Fig. 6A, B: testing apparatus and protocol, and 6C–F representative force-displacement curves). Cross-sectional tendon areas were measured and significantly larger values were obtained for the injured tendons compared to the controls, but no significant differences were detected between the genotypes (Fig. 5A). Next, dynamic Elastic modulus was measured at 3 different strain levels (4%, 6% and 8%) revealing significantly higher values at each deformation level in control *Tnmd*^*−/−*^ tendons compared to WT (Fig. 5B). Upon injury, dynamic E-modulus decreased considerably compared to non-injured controls, but a genotype difference was still detectable at 8% strain (Supplementary Fig. 6G). Furthermore, we evaluated the static E-modulus and yet again, the values in control *Tnmd*^*−/−*^ tendons were significantly higher than in the WT. At day 100 post-injury, this parameter showed significant and comparable 2-fold decrease in both genotypes (Fig. 5C). We also measured the load required to produce tissue failure, and found a significant difference towards lesser load inducing failure in the injured groups, but our biomechanical system did not detect significant changes between the control groups (Supplementary Fig. 6H). Lastly, similar to the static E-modulus, control *Tnmd*^−/−^ tendons exhibited a strong tendency (*p* = 0.0543) to be stiffer than WT tendons (Fig. 5D). Irrespective of genotype, injury led to significant loss of tissue stiffness at day 100 that might relate to the observed trauma-induced tendon HO at this stage. Because of the significantly impaired tendon biomechanical properties upon injury, as well as the differences in the contralateral Achilles tendons, we tested the tissue functionality by allowing *Tnmd*^*−/−*^ and WT mice to voluntarily undertake overnight running at day 100 post-injury (Fig. 5E). We detected a significantly lower running distance in *Tnmd*^*−/−*^ animals, which we attributed to their significantly stiffer and hence inefficient contralateral tendons compared to WTs (Fig. 5F). Altogether, we demonstrate a profound decline in tendon viscoelastic properties at the late stage of Achilles tendon healing concomitant with diminished running behavior of the *Tnmd*^*−/−*^ group; moreover, non-injured *Tnmd*-deficient tendons are stiffer and exhibit increased resistance to elastic deformation and reduced capacity to store muscle-generated energy. All in all, in Fig. 5G we provide a graphical synopsis of the major phenotypic changes in *Tnmd*-deficient mice over the time course after injury.

### Pilot single cell RNA-sequencing analysis reveals a significant transcriptomic shift between *Tnmd*^−^^*/−*^*ScxGFP*^*+*^ and WT *ScxGFP*^*+*^ cells

**Fig. 6:**
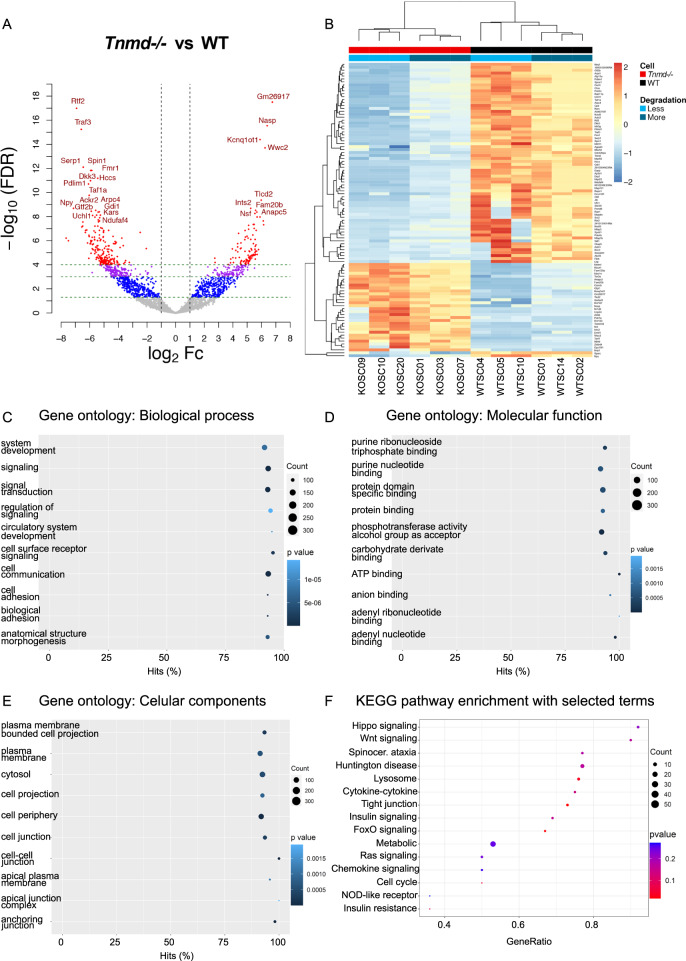
Single cell RNA-sequencing of *Tnmd*^*−/−*^ ScxGFP^+^ and WT ScxGFP^+^ cells isolated from non-injured Achilles tendons. **A** Volcano plot shows –Log_10_FDR i.e. adjusted p-value and Log_2_Fc expression values of all genes, each dot represents a gene. Significantly differentially expressed genes (DEGs) (–Log_10_FDR < 0.05 and |Log_2_ Fc | > 1) are highlighted in color other than gray. Vertical lines present the Log_2_Fc threshold equaling 1 or −1; genes outside these lines are DEGs with at least 2-Fc. The horizontal lines show –Log_10_FDR corresponding to FDR thresholds of 0.05, 0.001 and 0.0001 from the bottom respectively. Red, purple and blue dots represent the significant DEGs with 2-Fc and –Log_10_FDR < 0.0001, –Log_10_FDR < 0.001 and –Log_10_FDR < 0.05 respectively. Gray dots are neither significant nor have expression above 2-Fc. **B** Heat map depicting the Log_2_CPM expression value of selected top 100 significant DEGs. Top 100 genes with lowest q-value are depicted. Genes are colored according to their Log_2_CPM expression values. Clustering on the top is based on cell type and degradation level. **C**–**E** GO enrichment analysis of the significantly DEGs; results are depicted in dot plot format where top 10 enriched ontology terms are listed on *y*-axis and the percentage of hits (number of DEGs/number of background genes associated to each term) are indicated on x-axis. Dots size represents the count of DEGs associated with each term, whilst the color shows the enrichment *p*-value. Top enriched terms from **C** Biological Process, **D** Molecular Function and **E** Cellular Component. **F** KEGG signaling pathway analysis of the significant DEGs in dot plot format; *y*-axis shows top 15 enriched pathways; x-axis indicates gene ratio (number of DEGs/number of background genes associated to each term). Dots size is based on the number of DEGs that were associated with each term, whilst the color shows the *p*-value of enrichment.

To fish out potential transcriptomic alterations driven by the absence of *Tnmd* in tendon lineage cells, we subjected *Tnmd*^*−/−*^ ScxGFP^+^ and WT ScxGFP^+^ cells, isolated from non-injured Achilles tendons, to single cell RNA-sequencing (scRNA-seq) analysis. A total of 1278 significantly differentially expressed genes (DEGs) were identified between the genotypes by DESeq2-analysis. Volcano plot revealed that 529 DEGs were upregulated and 749 DEGs were downregulated in the *Tnmd*^*−/−*^ cells compared to the WTs (Fig. 6A). Heat map (Fig. 6B) of the top 100 significant DEGs pointed out gene targets such as ninjurin-1 (*Ninj1*), E2F-associated phosphoprotein *(Eapp)*, reticulocalbin-3 *(Rcn3)*, serpin H1 *(Serpinh1)*, spinlin-1 *(Spin1)* and secreted protein acidic and cysteine rich *(Sparc)*, to be significantly downregulated in *Tnmd*^*−/−*^ group. These genes are linked to regulatory mechanisms of cell adhesion, proliferation, apoptosis and collagen synthesis. In contrast, cyclin-D-binding Myb-like transcription factor 1 (*Dmtf1*), E3 ubiquitin-protein ligase *Rnfl130* and *Rnfl167*, glycosaminoglycan xylosylkinase (*Fam20b*) and the constitutive coactivator of PPAR-gamma-like protein 1 (*Fam120b*) were significantly upregulated in *Tnmd*^*−/−*^ cells (Fig. 6B, Supplementary Table 3). These genes play roles in negative regulation of cell cycle, senescence, proteoglycan expression and adipose cell differentiation. Gene ontology (GO) analysis (Fig. 6C–E) of only the significantly DEGs showed enrichment of gene clusters under biological process (BP), molecular function (MF) and cellular component (CC). For BP, the top 10 enriched terms included “signaling”, “cell surface receptor signaling pathway”, “cell adhesion”, “cell communication” and “circulatory system development” (Fig. 6C). For MF (Fig. 6D), “protein domain specific binding”, “phosphotransferase activity” and “purine and adenyl nucleotide binding” scored in the top 10 enriched terms. For CC, terms related to “cell periphery”, “plasma membrane bounded cell projection” and “anchoring and cell junction” were among the top 10 enriched terms (Fig. 6E). When subjecting all DEGs to Kyoto Encyclopedia of Genes and Genomes (KEGG) signaling pathway analysis, hits on “Hippo and Wnt-signaling cascades”, “metabolic and insulin signaling pathways”, and “chemokine and receptor signaling pathways” were uncovered (Fig. 6F). In sum, this very novel scRNA-seq data demonstrates that loss of *Tnmd* results in profound gene expression changes in tendon lineage cells from the non-injured Achilles tendon setting. It also suggests unprecedented signaling pathways involving indirectly or directly *Tnmd* and strongly urges for follow up analyses on tendon lineage cells from the injured setting.

## Discussion

HO is an aberrant repair process involving ectopic bone formation in response to tendon tissue trauma [16]. First, local tissue destruction and inflammation are initiated followed by activation and proliferation of fibroblastic cells, which in turn can differentiate into chondrocytes and osteoblasts, thereby resulting in HO with adverse effects to the tendon structure, strength and function [33]. Despite the clinical relevance of trauma-induced tendon HO [3, 4], the evolution of cell populations as well as the molecular mechanisms of HO have not been fully elucidated. Here, we challenged *Tnmd*-knockout mice with complete Achilles tenotomy and we hypothesized *Tnmd* to be a protective factor of trauma-induced HO in tendon based on: (i) loss of *Tnmd* results in augmented fibrovascular scar tissue during early tendon healing [26]; (ii) *Tnmd*-deficiency is associated with aging-related mineralization of the chordae tendineae cordis [34]; and (iii) double knockout of *Tnmd* and its homolog chondromodulin I (*Chm1*) leads to ectopic appearance of hypertrophic chondrocytes and osteoblasts in intervertebral disc [35].

Our results demonstrated that the absence of *Tnmd* alters the interplay between ScxGFP^+^ tendon lineage cells and αSMA-expressing myofibroblastic cells. We detected 8 days after injury only minor numbers of ScxGFP^+^ cells being located in the scar lesions, which is consistent with other studies [12, 36]. During the later repair stages, ScxGFP^+^ cells increased although their numbers were significantly less in *Tnmd*^*−/−*^ than WT scars. Tnmd might directly influence the tendon lineage cells, since it has been shown to regulate tendon cell density, proliferation and senescence in vivo and in vitro [20, 22, 26]. Interestingly, we detected significantly more αSMA^+^ cells in day 8 *Tnmd*^*−/−*^ scars. In healthy tendons, αSMA^+^ cells are contained in the vascularized epitenon sheaths [37], and upon injury they activate and dominate the fibrotic scar [12, 38]. We propose that the significant influx of αSMA^+^ cells in *Tnmd*^*−/−*^ scars is related to the enhanced vascularization of the ruptured mutant tendons [26]. Remarkably, we detected some double positive ScxGFP^+^/αSMA^+^ cells in the three different time points post-injury. Best and Loiselle proposed that Scx^+^ cells appear early in the scar tissue of injured and sutured tendons, but do not differentiate into myofibroblasts during healing [14] while several studies have suggested αSMA cell plasticity [39–42] including towards ScxGFP^+^ [13]. Next, in day 21 scars we detected cartilaginous anlage with a tendency to be larger in the *Tnmd*^*−/−*^ group. The chondrocyte origin is still disputable and several cell types can differentiate into this lineage, including Scx-expressing subpopulations: Scx^+^CD105^−^, Scx^+^αSMA^+^, Scx^+^S100a4^+^, Scx^+^Sox9^+^ and Cathepsin-K^+^ cells [43–46]. Whether these discrete subpopulations have been altered upon *Tnmd* loss should be addressed in follow up research. Macrophages have a pivotal task in both stimulating and resolving inflammation, therefore assisting and regulating the ongoing tissue repair [7]. The phenotype reported at day 8 [26] was persistent also at day 21, suggesting that *Tnmd* absence can result in a chronic inflammation in the injured tendons. A study by Sorkin et al. revealed in burn tenotomy that invading macrophages drive tendon HO by producing transforming growth factor beta-1 (TGF-ß1), thus stimulating mesenchymal progenitors to chondrogenically differentiate [16]. Indeed, at day 8 significantly *Tgf-ß1* mRNA levels were increased in tendon tissue extracts from *Tnmd*^*−/−*^ group [26]. The molecular mechanisms involved in the crosstalk between distinct cell populations during trauma-induced tendon HO still remains to be unraveled. To this end, our data provides the first evidences that *Tnmd* might be an important molecular factor involved in the interplay between cellular populations during tendon HO process.

We followed the repair process up to day 100 after injury, and we monitored ECM fibrillar composition by TEM, HO spreading by µCT and biomechanical properties by viscoelastic testing. Our TEM data validated a very consistent ECM phenotypic feature specific to the loss of *Tnmd* [20, 24], namely a significant increase of COL fibril diameters coupled with aberrant fibril morphology. At the ultrastructural level mineral deposits and osteocyte-like cells were observed in both genotypes at day 100 post-trauma; however, µCT-based quantification revealed a significantly higher HO in *Tnmd*^*−/−*^ tendons compared to WT. Biomechanical analysis showed that even after 100 days of injury, the tendons in both groups suffer significant negative changes in their viscoelastic properties compared to non-injured tendons. We detected only significant differences in injured tendons in the dynamic E-modulus at 8% strain, which might be related to the sensitivity of the biomechanical testing system when HO-compromised tendons were measured. However, we discovered in non-injured controls as well as in the contralateral tendons of injured animals that the absence of *Tnmd* results in a significant increase in static and dynamic E-moduli, representing Achilles tendon stiffening. These findings correlate with the pathological thickening of COL fibrils [20, 24] and increase in their nano-stiffness [24] as well as dysregulated expression of genes regulating COL fibrilogenesis in *Tnmd*^*−/−*^ tendons [26]. Biomechanical alteration in the tendon tissue, serving as a spring in the locomotive apparatus can result in deficient storage and transmission of the muscle-generated energy, hence reduced tendon functionality. We have shown that *Tnmd*^*−/−*^ mice ran significantly less, experienced fatigue and developed secondary myopathy [24]. In this study, we subjected at day 100 post-injury *Tnmd*^*−/−*^ animals to voluntary running tests and found that, again, this group undertakes significantly shorter distances compared to the WTs. We propose that this behavior is the combined result of the significant stiffening of the contralateral Achilles tendons and significant HO of the injured ones.

Altogether, our results on the scar tissue composition and the data on qRT-PCR at day 8 [26], 21 and 100 can also be interpreted as the two study groups having an uneven healing baseline: the speed of the healing process in *Tnmd*^*−/−*^ group was delayed, while the WT group had more advanced scar remodeling. To clarify this hypothesis future investigations should assess time points later than 100 days to determine whether WTs reach full remodeling, while mutants maintain tendon HO. Other relevant research approaches to consider are: evaluating an earlier cerclage removal thus enabling sooner load transmission; allowing animals to run in order to facilitate further remodeling; utilizing Tnmd overexpression mouse model to assess if healing can accelerate; and employing a commercially available recombinant Tnmd protein as a rescue trial.

Due to the lack of knowledge on direct binding partners of *Tnmd*, a major limitation of our study is the explanation of the molecular mode-of-action of this protein. Our scRNA-Seq analysis revealed clear genotype differences and suggested that loss of *Tnmd* results in dysregulated gene expression associated with cell adhesion, proliferation, senescence and collagen synthesis, the latter based on the significant downregulation of *Rcn3* and *Serpinh1* [47, 48]. Among the enriched GO clusters, the frequently referenced genes belong to bone morphogenic protein family. KEGG signaling pathway analysis identified Hippo-signaling pathway, connected to the transcriptional co-activator complex YAP/TAZ that plays key roles in tissue homeostasis and regeneration. Remarkably, elevated BMP- and Hippo-related signaling have been strongly allied with HO of mesenchymal progenitors [49]. Another KEGG hit was Wnt-pathway, which is in line with in vitro studies reporting that Wnt/ß-catenin signaling can mediate Scx-independent expression of *Tnmd* in tendon- and bone marrow-derived mesenchymal stem cells [50, 51]. KEGG analysis also referenced metabolic and insulin pathways, which is in agreement with genetic, clinical and experimental studies that have linked *Tnmd* to metabolic syndrome, diabetes and obesity [19]. In all, the scRNA-seq approach can support the identification of putative *Tnmd*-dependent cascades and elucidation of *Tnmd* mode-of-action; hence, follow up studies should aim to screen and validate possible molecular targets and pathways as well as implement protein-protein interaction assays. Moreover, by employing scRNA-seq on tendon lineage cells isolated from distinct stages of the repair process, a better understanding of the longitudinal molecular “evolution” in the Achilles tendons from both genotypes can be gained.

Taken together, our study demonstrates that loss of *Tnmd* leads to long-term inferior tendon healing outcome characterized by: i) imbalance between tendon lineage and myofibroblastic cells during early stage; ii) persistent inflammatory macrophage profile and emergence of robust cartilaginous template during the proliferative stage; and iii) significant in-tendinous heterotopic ossification concurrent with compromised tendon viscoelastic properties and reduced running capacity at the remodeling stage (graphical synopsis, Fig. 5G). Thus, we report that *Tnmd* plays an important role during the course of tendon healing by alleviating trauma-induced HO and, therefore, is a suitable gene candidate for the generation of novel dual function therapeutic drugs—accelerating repair and reducing the risk of undesired ossification of injured tendons.

## Supplementary information

Supplementary Information

Supplementary Fig. 1

Supplementary Fig. 2

Supplementary Fig. 3

Supplementary Fig. 4

Supplementary Fig. 5

Supplementary Fig. 6

Surgery Video

## Data Availability

The authors declare that the datasets generated and/or analyzed during the current study are available from the corresponding author on reasonable request. The scRNA-Seq datasets used for the analysis have been deposited into the Gene Expression Omnibus database at the National Center for Biotechnology Information (Accession number GSE179454).
