# Supplementary material for: Tenomodulin knockout mice exhibit worse late healing outcomes with augmented trauma-induced heterotopic ossification of Achilles tendon
Source: Cell Death Dis. 2021 Nov 5;12(11):1049. doi: 10.1038/s41419-021-04298-z (PMC8571417; doi:10.1038/s41419-021-04298-z)
Supplement: Supplementary file 1 — Supplementary Information [file 41419_2021_4298_MOESM1_ESM.docx]

Supplementary Information for

**Tenomodulin knockout mice exhibit worse late healing outcomes with augmented trauma-induced heterotopic ossification of Achilles tendon**

Manuel Delgado Caceres, et al.

*Corresponding author. Email: denitsa.docheva@ukr.de

**___________________________________________________________________________**

**The PDF file includes:**

**Supplementary Figure Legends**

**Supplementary Figure 1:** Experimental design and surgical procedure

**Supplementary Figure 2:** Analysis of vessels, nerves and pericytes at day 21 post-injury

**Supplementary Figure 3:** Scar organization, collagen fibers alignment and gene expression analysis

**Supplementary Figure 4:** Transmission electron microscopy and collagen fibril diameter distribution in contralateral, non-injured tendons

**Supplementary Figure 5:** Micro-CT analyses for age-related HO and bone quality of hindlimbs

**Supplementary Figure 6:** Biomechanical testing

**Supplementary Table 1:** Histological scoring system, 21 and 100 days post-injury

**Supplementary Table 2:** TEM fibril diameter quantification

**Supplementary Table 3:** Top 100 differentially expressed genes based on q-values

**Supplementary Material and Methods**

___________________________________________________________________________

**Supplementary Figure Legends**

**Supplementary Fig. 1. Experimental design and surgical procedure.** (A) Experimental group design. (B) Representative images of the surgical procedure. (C) Representative macroscopic images of explanted *Tnmd^-/-^* and WT hindlimbs at 8, 21 and 100 days post-injury. Scale bar: 50mm

**Supplementary Fig. 2.** **Analysis of vessels, nerves and pericytes at day 21 post-injury.** (A, B) Representative mosaic, (A1, B1) high magnification DAB images of Neurofilament heavy polypeptide (NEFH) staining and (C) quantification of the positive area/tendon. (D, E) Representative mosaic, (D1, E1) high magnification fluorescent images of collagen IV (COLIV) staining and (F) quantitative analysis of ColIV^+^ blood vessels/tendon. (G) Histological scoring at day 21. (H, I) Representative mosaic, (H1, I1) high magnification fluorescent images of the pericyte marker CD146 and (J) quantitative analysis of CD146^+^ cells within tendon scar tissue. Black and white dotted lines frame Achilles tendon areas. C=calcaneus, TP=tendon proper, S=skin. Scale bar: 200µm (panels A-I); 50µm (panel A1-I1), 20µm (zoom in from panels D1, E1, H1 and I1). Histological scoring, n=10 animals/genotype; stainings, n=4-6 animals/genotype, each animal was represented by three tissue sections. Box plots show median + interquartile range (IQR). Empty dot represents female mouse; filled dot represents male mouse.

**Supplementary Fig. 3.** **Scar organization, collagen fibers alignment and gene expression analysis.** (A, B) Representative and (A1, B1) high magnification images of Herovici staining. (C, D) Representative mosaic, (C1, D1) high magnification images of Picrosirius red staining. Yellow dotted lines frame Achilles tendon areas. C=calcaneus. Scale bar: 200µm (panels A-D); 50µm (panel A1-D1). Stainings were performed with n=4-6 animals/genotype, each animal was represented by three tissue sections. (E) List of well-known tendon-related gene markers. GF=Growth factor, TF=transcription factor, ECM=Extracellular matrix. (F) qRT-PCR analysis at day 21 and (G) at day 100 post-injury. Pool of n=3 injured Achilles tendons/genotype/time point, 3 independent experiments. Statistical significance was calculated using two-tailed, unpaired parametric Student’s t-test. **p<0.01, ***p<0.001, ****p>0.0001.

**Supplementary Fig. 4. Transmission electron microscopy and collagen fibril diameter distribution in contralateral, non-injured tendons.** (A-D) Representative 10000x and (A1-D1) 40000x magnification images of contralateral *Tnmd^-/-^* and WT Achilles tendons. T=Tenocyte; yellow arrow=cell protrusions. Scale bars: 2µm (panels A-D); 500nm (panels A1-D1). (A2-D2) Histograms showing incidence of collagen fibril size.(E) Violin plot for collagen fibril diameter distribution, median + IQR, statistical significance was calculated by 2-tailed unpaired nonparametric Mann-Whitney test, *p<0.05, **p<0.01, ***p<0.001, ****p<0.0001. Day 21, n=2 animals/genotype; day 100 n=2 animals/genotype.

**Supplementary Fig. 5.** **Micro-CT analyses for age-related HO and bone quality of hindlimbs.** (A, B) Representative µCT scan of 9-months old contralateral and non-injured, control *Tnmd^-/-^* and WT Achilles tendons (HO marked in red and green color). Total HO volume (C) and surface (D) quantification. (E) Representative µCT scan of calcaneal and tibiofibular bones. Calcaneal bone volume (F) and surface (G) (magenta region quantified). Tibiofibular bone volume (H) and surface (I) (blue region quantified). n=4-8 animals/genotype. Box plots show median + IQR. Statistical analysis was performed with one-way ANOVA. Empty dot represents female mouse; filled dot represents male mouse.

**Supplementary Fig. 6.** **Biomechanical testing.** (A) Biomechanical testing set up. (B) Viscoelastic biomechanical protocol: step 1, preconditioning; step 2, hold; step 3, increment to 4% strain and frequency sweep at 0.01, 0.1, 1, 5 and 10 Hz; step 4, increment to 6% strain; step 5, increment to 8%; step 6, return to preload displacement, step 7, ramp to failure at 0.1%/s. Representative force-displacement curves for control (C, D) and injured *Tnmd^-/-^* and WT Achilles tendons (E, F). (G) Dynamic E-modulus of injured *Tnmd^-/-^* and WT tendons (day 100) at three different strain levels. (H) Load-to-failure tests. Box plots show median + IQR. Statistical analysis was performed with one-way ANOVA across genotype with Bonferroni post hoc tests, *p<0.05, **p<0.01, ***p<0.001, ****p<0.0001. n=8-14 animals/genotype. Empty dot represents female mouse; filled dot represents male mouse.

**Supplementary Table 1.** Histological scoring system, 21 and 100 days post-injury

| Evaluated parameters | Points |
| --- | --- |
| *Extracellular matrix (ECM) organization of the whole tendon*  Wavy, compact and parallel arranged collagen fibers  In part compact, in part loose or not orderly  Loosely composed, not orderly (“granulation” tissue) | 2  1  0 |
| *Cellularity/cell-matrix-ratio*  Physiological  Locally increased cell density  Increased cell density or decreased ECM content | 2  1  0 |
| *Cell alignment*  Uniaxial  Areas of irregularly arranged cells (10-50%)  More than 50% of cells with no uniaxial alignment | 2  1  0 |
| *Cell distribution*  Homogeneous, physiological  Focal areas of elevated cell density (cell clustering) | 1  0 |
| *Cell nucleus morphology*  Predominantly elongated, heterochromatic cell nuclei (tenocytes)  10-30% of the cells possess large, oval, euchromatic or polymorph heterochromatic nuclei  Predominantly larger, oval, euchromatic or polymorph, heterochromatic nuclei | 2  1  0 |
| *Organization of repair tissue of the tendon callus*  Homogeneous (whole tissue with similar composition)  Locally heterogeneous tissue composition  Whole tissue composition completely changed | 2  1  0 |
| *Transition from defect to normal tissue*  Scaffold integrated, no gaps at the margin visible  Recognizable transition  Abrupt transition, splitting/gaps detectable, callus tissue | 2  1  0 |
| *Configuration of callus*  Normal, only in the defect area, locally confined  Strong, change of whole tendon, thickened | 1  0 |
| *Cartilage formation (COL II staining)*  Non existing  Focally increased  Assembly of cartilage (more than 25% of the tendon) | 2  1  0 |
| *Degenerative changes/tissue metaplasia*  Non existing  Moderate formation of oedema  Intense oedema with inclusion of fat, cell and/or fibers destruction, fibrin deposition, gaps, adhesions | 2  1  0 |
| *Inflammation (CD68/CD163 staining)*  No inflammatory cell infiltrates  Infiltrating inflammatory cells (macrophages) | 1  0 |
| *Vascularization in the defect area (COL IV staining)*  Hypo-vascularized, like surrounding tendon (small capillaries)  Hyper-vascularized (increased numbers of small or larger capillaries) | 1  0 |
| *Nerves (NEFH-staining)*  Non existing  Focally increased (less than 25% of the entire tendon length)  Distributed through/along the entire tendon (more than 25%) | 2  1  0 |
| *Bone formation (OPN staining)* 🡪 Analized only 100 days post-injury  Non existing  Focally increased (less than 25% of the tendon length)  Distributed through/along the entire tendon (more than 25%) | 2  1  0 |

References [1, 2]

**Supplementary Table 2.** TEM fibril diameter quantification

| Statistic | **Injured tendons day 21** | | **Injured tendons day 100** | |
| --- | --- | --- | --- | --- |
|  | *Tnmd^-/-^* | WT | *Tnmd^-/-^* | WT |
| Total number of values | 4575 | 5088 | 4832 | 6021 |
| Minimum | 1.64 | 1.16 | 2.32 | 2.36 |
| Median | 32.93 | 35.60 | 49.26 | 34.74 |
| Maximum | 332.87 | 278.06 | 2.95 | 220.31 |
| Mean | 48.28 | 39.19 | 57.54 | 40.72 |
| SD | 50.51 | 25.93 | 39.91 | 27.80 |
| SEM | 0.75 | 0.36 | 0.57 | 0.36 |
|  | | | | |
| Statistic | **Contralateral tendons day 21** | | **Contralateral tendons day 100** | |
|  | *Tnmd^-/-^* | WT | *Tnmd^-/-^* | WT |
| Total number of values | 1488 | 1857 | 1127 | 1846 |
| Minimum | 33.16 | 12.47 | 35.05 | 5.18 |
| Median | 150.00 | 125.80 | 147.60 | 113.45 |
| Maximum | 277.00 | 229.60 | 293.85 | 246.46 |
| Mean | 148.30 | 122.30 | 145.83 | 109.90 |
| SD | 41.67 | 41.36 | 52.96 | 42.37 |
| SEM | 1.08 | 0.96 | 1.58 | 0.99 |

**Supplementary Table 3.** Top 100 differentially expressed genes based on q-values

| **Upregulated genes in *Tnmd^-/-^*** | **Base Mean** | **log_2_ Fold change** | **lfcSE** | **p-value** | **q-value** | **logcpm.**  ***Tnmd^-/-^*** | **logcpm.**  **WT** |
| --- | --- | --- | --- | --- | --- | --- | --- |
| Gm26917 | 582.48 | 6.755 | 0.702 | 6.75E-22 | 3.16E-18 | 7.339 | 0.719 |
| Nasp | 1910.93 | 6.399 | 0.709 | 1.86E-19 | 2.91E-16 | 8.882 | 2.350 |
| Kcnq1ot1 | 541.44 | 5.899 | 0.681 | 4.36E-18 | 4.09E-15 | 7.297 | 1.437 |
| Wwc2 | 335.35 | 6.250 | 0.738 | 2.55E-17 | 1.99E-14 | 6.524 | 0.490 |
| Tlcd2 | 649.66 | 5.982 | 0.843 | 1.30E-12 | 4.34E-10 | 7.097 | 1.252 |
| Ints2 | 131.53 | 5.245 | 0.765 | 7.05E-12 | 2.07E-09 | 5.104 | 0.167 |
| Fam20b | 433.1 | 5.593 | 0.824 | 1.13E-11 | 3.03E-09 | 6.689 | 1.206 |
| Anapc5 | 546.33 | 5.677 | 0.846 | 1.95E-11 | 4.25E-09 | 6.888 | 1.364 |
| Nsf | 216.68 | 5.541 | 0.832 | 2.72E-11 | 5.54E-09 | 5.567 | 0.547 |
| Gstm5 | 721.90 | 5.897 | 0.902 | 6.26E-11 | 1.09E-08 | 6.829 | 1.486 |
| Ikbkb | 211.71 | 5.695 | 0.872 | 6.52E-11 | 1.09E-08 | 5.341 | 0.387 |
| Polr1a | 228.17 | 6.126 | 0.958 | 1.63E-10 | 2.31E-08 | 5.113 | 0.014 |
| Tomm34 | 244.62 | 5.431 | 0.866 | 3.56E-10 | 4.52E-08 | 5.652 | 0.716 |
| Myo1e | 389.92 | 5.488 | 0.875 | 3.57E-10 | 4.52E-08 | 6.247 | 1.215 |
| Nr1d2 | 351.18 | 6.153 | 0.983 | 3.92E-10 | 4.83E-08 | 5.325 | 0.630 |
| Terf1 | 213.76 | 5.103 | 0.818 | 4.48E-10 | 5.39E-08 | 5.688 | 0.826 |
| Dmtf1 | 207.25 | 5.486 | 0.896 | 9.36E-10 | 9.64E-08 | 5.381 | 0.364 |
| Fam120a | 282.76 | 5.101 | 0.838 | 1.13E-09 | 1.13E-07 | 6.065 | 1.112 |
| Usp24 | 227.39 | 5.628 | 0.932 | 1.56E-09 | 1.41E-07 | 5.107 | 0.526 |
| Sertad1 | 796.94 | 4.854 | 0.805 | 1.66E-09 | 1.44E-07 | 7.523 | 2.554 |
| Tor3a | 255.36 | 4.400 | 0.743 | 3.24E-09 | 2.49E-07 | 6.117 | 1.806 |
| Gfpt1 | 490.71 | 5.396 | 0.920 | 4.54E-09 | 3.42E-07 | 6.576 | 1.254 |
| Zbtb44 | 103.00 | 4.988 | 0.858 | 5.99E-09 | 4.32E-07 | 4.321 | 0.063 |
| Metrnl | 279.00 | 4.876 | 0.844 | 7.58E-09 | 5.07E-07 | 6.034 | 1.277 |
| Rnf130 | 89.19 | 5.064 | 0.883 | 9.93E-09 | 6.20E-07 | 4.032 | -0.230 |
| Nrip1 | 213.24 | 5.248 | 0.923 | 1.32E-08 | 7.95E-07 | 5.189 | 0.647 |
| Dpy19l1 | 156.28 | 5.557 | 0.978 | 1.32E-08 | 7.95E-07 | 4.280 | 0.094 |
| Rbck1 | 252.59 | 4.835 | 0.853 | 1.44E-08 | 8.33E-07 | 5.872 | 1.162 |
| Rnf167 | 711.52 | 4.564 | 0.825 | 3.21E-08 | 1.56E-06 | 7.388 | 2.576 |
| Arl4d | 170.51 | 5.436 | 0.989 | 3.82E-08 | 1.78E-06 | 4.340 | 0.3269 |

| **Downregulated genes in *Tnmd*^-/-^ cells** | **Base Mean** | **log_2_ Fold change** | **lfcSE** | **p-value** | **q-value** | **logcpm.**  ***Tnmd^-/-^*** | **logcpm.**  **WT** |
| --- | --- | --- | --- | --- | --- | --- | --- |
| Rtf2 | 673.49 | -6.921 | 0.734 | 4.39E-21 | 1.03E-17 | 0.927 | 7.412 |
| Traf3 | 372.32 | -6.589 | 0.739 | 4.97E-19 | 5.82E-16 | 0.501 | 6.741 |
| Serp1 | 338.11 | -6.451 | 0.805 | 1.19E-15 | 7.95E-13 | 0.474 | 6.380 |
| Fmr1 | 246.61 | -5.854 | 0.740 | 2.77E-15 | 1.48E-12 | 0.532 | 6.186 |
| Spin1 | 249.23 | -5.926 | 0.750 | 2.84E-15 | 1.48E-12 | 0.525 | 6.153 |
| Hccs | 1417.19 | -5.438 | 0.706 | 1.41E-14 | 6.60E-12 | 0.173 | 5.494 |
| Dkk3 | 479.12 | -5.947 | 0.780 | 2.49E-14 | 1.06E-11 | 1.268 | 6.900 |
| Pdlim1 | 502.44 | -6.087 | 0.808 | 5.08E-14 | 1.98E-11 | 1.197 | 6.826 |
| Taf1a | 388.03 | -6.035 | 0.834 | 4.82E-13 | 1.74E-10 | 0.914 | 6.468 |
| Npy | 15199.1 | -7.134 | 1.041 | 7.01E-12 | 2.07E-09 | 4.252 | 10.859 |
| Arpc4 | 321.6 | -5.580 | 0.822 | 1.16E-11 | 3.03E-09 | 1.008 | 6.301 |
| Ackr2 | 1016.31 | -5.889 | 0.873 | 1.48E-11 | 3.65E-09 | 1.616 | 7.681 |
| Gdi1 | 147.05 | -5.326 | 0.792 | 1.74E-11 | 4.08E-09 | 0.321 | 5.267 |
| Gtf2b | 364.41 | -5.414 | 0.807 | 2.00E-11 | 4.25E-09 | 1.333 | 6.493 |
| Ndufaf4 | 196.56 | -5.256 | 0.796 | 3.95E-11 | 7.64E-09 | 0.807 | 5.659 |
| Kars | 453.79 | -5.515 | 0.835 | 4.08E-11 | 7.64E-09 | 1.542 | 6.611 |
| Uchl1 | 688.99 | -5.797 | 0.880 | 4.52E-11 | 8.15E-09 | 1.573 | 6.974 |
| Ptrhd1 | 286.82 | -5.634 | 0.864 | 6.95E-11 | 1.12E-08 | 0.880 | 5.871 |
| Rab11a | 495.35 | -5.301 | 0.816 | 8.04E-11 | 1.26E-08 | 1.767 | 6.834 |
| Drg1 | 194.98 | -5.405 | 0.840 | 1.25E-10 | 1.89E-08 | 0.547 | 5.491 |
| Eapp | 211.21 | -5.314 | 0.831 | 1.60E-10 | 2.31E-08 | 0.764 | 5.631 |
| Ap3d1 | 192.97 | -5.388 | 0.847 | 2.01E-10 | 2.77E-08 | 0.535 | 5.446 |
| Myadm | 342.73 | -6.478 | 1.019 | 2.12E-10 | 2.84E-08 | 0.307 | 5.207 |
| Psmd6 | 334.28 | -6.491 | 1.046 | 5.35E-10 | 6.22E-08 | 0.424 | 5.001 |
| Cpd | 425.61 | -5.634 | 0.908 | 5.54E-10 | 6.22E-08 | 1.212 | 6.296 |
| Idh3g | 292.29 | -5.434 | 0.876 | 5.58E-10 | 6.22E-08 | 1.076 | 5.872 |
| Spsb1 | 466.41 | -6.551 | 1.061 | 6.74E-10 | 7.34E-08 | 0.795 | 5.110 |
| Ninj1 | 290.07 | -5.225 | 0.850 | 8.01E-10 | 8.53E-08 | 1.132 | 6.076 |
| Dcun1d1 | 277.72 | -5.128 | 0.838 | 9.46E-10 | 9.64E-08 | 1.191 | 6.073 |
| Ssr2 | 202.30 | -6.208 | 1.024 | 1.36E-09 | 1.32E-07 | -0.031 | 4.665 |
| Ckap5 | 318.08 | -6.290 | 1.040 | 1.47E-09 | 1.40E-07 | 0.404 | 4.962 |
| Spire1 | 297.79 | -5.320 | 0.880 | 1.53E-09 | 1.41E-07 | 1.063 | 5.978 |
| Mrpl23 | 111.66 | -4.676 | 0.774 | 1.55E-09 | 1.41E-07 | 0.572 | 4.879 |
| Kctd9 | 374.88 | -5.024 | 0.834 | 1.67E-09 | 1.44E-07 | 1.691 | 6.411 |
| AA467197 | 1112.57 | -5.702 | 0.949 | 1.84E-09 | 1.57E-07 | 2.059 | 7.057 |
| Map7d1 | 127.59 | -5.036 | 0.842 | 2.22E-09 | 1.86E-07 | 0.456 | 4.838 |
| 0610030E20Rik | 194.28 | -5.036 | 0.845 | 2.53E-09 | 2.08E-07 | 0.876 | 5.461 |
| Sparc | 10319.9 | -2.478 | 0.416 | 2.58E-09 | 2.09E-07 | 8.897 | 11.457 |
| Cfl2 | 278.08 | -5.606 | 0.942 | 2.65E-09 | 2.10E-07 | 0.817 | 5.490 |
| Nudt5 | 372.47 | -6.302 | 1.059 | 2.70E-09 | 2.11E-07 | 0.606 | 5.032 |
| Reps1 | 170.30 | -5.319 | 0.908 | 4.60E-09 | 3.42E-07 | 0.520 | 4.944 |
| Rae1 | 250.22 | -6.052 | 1.034 | 4.89E-09 | 3.58E-07 | 0.356 | 4.729 |
| 2810004N23Rik | 79.29 | -4.369 | 0.752 | 6.28E-09 | 4.45E-07 | 0.372 | 4.401 |
| Rcn3 | 300.87 | -6.036 | 1.040 | 6.56E-09 | 4.59E-07 | 0.710 | 4.863 |
| 1600012H06Rik | 274.64 | -5.048 | 0.871 | 6.79E-09 | 4.67E-07 | 1.218 | 5.907 |
| Serpinh1 | 182.81 | -5.669 | 0.981 | 7.53E-09 | 5.07E-07 | 0.275 | 4.681 |
| Mllt11 | 654.86 | -5.828 | 1.010 | 7.95E-09 | 5.25E-07 | 1.537 | 5.965 |
| Atp11a | 257.95 | -5.062 | 0.878 | 8.14E-09 | 5.29E-07 | 1.144 | 5.760 |
| Ctsa | 568.05 | -5.328 | 0.929 | 9.85E-09 | 6.20E-07 | 1.745 | 6.500 |
| Nfe2l2 | 702.01 | -3.754 | 0.659 | 9.91E-09 | 6.20E-07 | 3.8301 | 7.600 |
| Wif1 | 4558.33 | -7.649 | 1.335 | 1.01E-08 | 6.21E-07 | 2.590 | 6.727 |
| Avpi1 | 341.57 | -5.222 | 0.919 | 1.34E-08 | 7.96E-07 | 1.368 | 5.911 |
| Stk38l | 195.88 | -5.658 | 0.996 | 1.36E-08 | 7.96E-07 | 0.476 | 4.658 |
| Rin2 | 243.44 | -5.653 | 0.999 | 1.51E-08 | 8.65E-07 | 0.682 | 4.902 |
| Flnb | 134.58 | -5.455 | 0.968 | 1.75E-08 | 9.90E-07 | -0.080 | 4.399 |
| Ufc1 | 182.59 | -5.458 | 0.969 | 1.79E-08 | 9.90E-07 | 0.469 | 4.708 |
| Pde4b | 324.32 | -5.922 | 1.052 | 1.80E-08 | 9.90E-07 | 0.893 | 4.812 |
| Polr2c | 555.64 | -5.268 | 0.936 | 1.85E-08 | 1.01E-06 | 1.714 | 6.480 |
| Cxcl1 | 521.29 | -5.034 | 0.896 | 1.91E-08 | 1.02E-06 | 1.901 | 6.616 |
| Cilp | 4211.69 | -7.490 | 1.333 | 1.94E-08 | 1.02E-06 | 2.500 | 6.781 |
| Jtb | 255.29 | -5.450 | 0.970 | 1.96E-08 | 1.02E-06 | 0.887 | 5.181 |
| Azin1 | 260.00 | -5.848 | 1.042 | 1.97E-08 | 1.02E-06 | 0.530 | 4.682 |
| 2410131K14Rik | 246.97 | -5.895 | 1.055 | 2.29E-08 | 1.18E-06 | 0.537 | 4.533 |
| Cdc42bpa | 118.23 | -4.225 | 0.756 | 2.35E-08 | 1.20E-06 | 0.783 | 5.062 |
| Abcf3 | 196.55 | -5.916 | 1.063 | 2.59E-08 | 1.30E-06 | 0.168 | 4.261 |
| Mfap1b | 104.72 | -4.919 | 0.884 | 2.67E-08 | 1.33E-06 | 0.224 | 4.338 |
| Trim8 | 107.75 | -4.143 | 0.746 | 2.81E-08 | 1.39E-06 | 0.960 | 4.880 |
| Mfap5 | 528.18 | -6.150 | 1.112 | 3.23E-08 | 1.56E-06 | 1.152 | 4.822 |
| Agpat4 | 1128.05 | -4.191 | 0.761 | 3.67E-08 | 1.76E-06 | 3.595 | 8.112 |
| Mrpl50 | 137.74 | -4.335 | 0.788 | 3.74E-08 | 1.77E-06 | 1.046 | 5.119 |

Abbreviations: LfcSE, log fold change Standard Error; mnlogcpm, mean value of log counts per million

**Supplementary Material and Methods**

**Animal model and surgical procedure.**

*Tnmd^-/-^* mice and WT littermates, as well as *ScxGFP* mice were previously described by Docheva et al. [3] and Pryce et al. [4], respectively. *Tnmd* gene is located on the X-Chromosome, therefore the WT group comprised hemizygous male, homozygous-, and heterozygous female mice. All analysed animals were maintained on the C57BL/6J background. The experimental design and the group distribution including n-numbers are shown in Supplementary Fig. 1A. Animals aged 6-months were selected because skeletal growth plateau was reached at this stage [5] and it corresponds to approx. 30-year old humans, which belong to the risk group for Achilles tendon rupture [6]. Mice were operated according to Lin et al. [2]. In brief, after anaesthesia and skin incision, the left Achilles tendon was fully resected 5 mm proximal of the calcaneus, followed by end-to-end reconstruction by Kirchmayr-Kessler suture technique (8-0 Ethilon) (Supplementary Fig. 1B). In order to avoid suture failure, the range of movement of the talocrural joint was restricted by a cerclage that was inserted through the tibiofibular fork and fixed between the calcaneus and the plantar aponeurosis (6-0 Prolene). This assures a limited degree of extension (~30%) but allowing tensile load transmission (Supplementary Fig. 1B). Representative macroscopic images at day 8, 21 and 100 post-injury are shown in Supplementary Fig. 1C.

Random assignment was used as randomization method. Surgical team was blinded to the experimental group allocation.

**Immunohistochemistry.** Mouse hindlimbs (exact n-numbers shown as dot plot as well as given in figure legends) were fixed overnight in fresh 4% paraformaldehyde (PFA, Merck, Darmstadt, Germany) in phosphate-buffered saline (PBS; pH 7.4) or in 95% ethanol- 5% glacial acetic acid. Specimens were decalcified in 10% ethylen diamine tetraacetic acid (EDTA)/phosphate buffered saline (PBS) pH 8.0 (Sigma-Aldrich, Munich, Germany) for 4 weeks, embedded in cryoprotective media and sectioned at 10µm, every 10^th^ slide was stained with Hematoxylin-Eosin following standard protocol. Tissue sections with equivalent regional planes between genotypes were selected for detailed investigation. Histological scoring was carried out by two independent observers at day 21 (n=10 animals/genotype) and 100 (n=11 animals/genotype) according to Stoll et al.[1] (Supplementary Table 1). To assess collagen maturation, Herovici staining (blue dye stains immature type III collagen, the red dye stains mature, organized type I collagen [7] was performed with Herovici collagen differentiation staining kit, (Morphisto, Frankfurt am Main, Germany) according to the manufacture’s protocol. Picrosirius red staining was performed using Direct Red 80 solution (Sigma-Aldrich) following standard protocol. Bright yellow to orange/red color indicates bigger and better aligned fibrils, while green color represents thin and poorly aligned fibrils.

For immunohistochemistry, tissue sections were treated with 10% H_2_O_2_ in methanol for 30 min, 1 mg/ml Pepsin (Sigma-Aldrich) for 15 min and blocked with 10% goat serum (Sigma-Aldrich) for 90 min (all at RT). Primary antibody against collagen type II (ab34712) and neurofilament-heavy chain (ab8135) (both from Abcam, Cambridge, UK) was diluted 1:50 in blocking solution and applied overnight at 4°C. Next day, corresponding goat anti rabbit biotinylated secondary antibody (Jackson ImmunoResearch, Pennsylvania, USA) was diluted 1:500 in blocking solution and applied for 1 hour at RT. Sections were treated with ABC-Kit (Vector Laboratories, Burlingame, USA) for 90 min, then with chromogen 3,3-diaminobenzidine tetrahydrochloride (DAB) and a nickel enhancer (Sigma-Aldrich) and mounted with DePeX (Serva, Heidelberg, Germany). In order to detect proliferative cells, 90 min. prior to euthanasia, operated mice received an intraperitoneal injection with BrdU (50 µg/g body weight, CatNr. 550891, BD Pharmingen, Franklin Lakes, New Jersey, USA), and tissue sections were stained as described above with BrdU-POD (REF11585860001, Sigma-Aldrich).

For immunofluorescence, tissue sections were treated with 2mg/ml hyaluronidase (Sigma-Aldrich) for 30 min at 37°C and subsequently blocked with 3% BSA bovine serum albumin (BSA)/PBS (Sigma-Aldrich) for 90 min. Primary antibodies against αSMA (ab5694), CD68 (ab125212), CD163 (ab182422), OPN (ab8448), collagen IV (ab6586) and CD146 (ab75769) (all Abcam) were applied overnight at 4°C, followed by goat anti-rabbit Cy3-conjugated secondary antibody (Jackson ImmunoResearch) for 1 hour at RT. Last, sections were counter-stained with 4’,6-diamidino-2-phenylindole (DAPI) for 10 min and finally mounted.

In order to quantify positively labeled cells (ScxGFP, αSMA, CD68, and CD163), an automated quantitative image analysis was performed using ImageJ, software version 1.8.0_112 (National Institutes of Health, Bethesda, MD, USA). The following algorithm was applied: (1) region of interest (ROI) was manually designated from mosaic images (each animal was represented by three different tissue sections) of antibody-stained sections using the “freehand selection” tool; (2) RGB image was converted to 8-bit gray scale and inverted; (3) threshold for black and white colors was accustomed and fixed; (4) “Image-based Tool for Counting (ITCN)” was adjusted; (5) using the “analyze tool” the particles were automatically counted; (6) the procedure was applied first to quantify the total number of DAPI^+^ nuclei, and afterwards with the green and red fluorescence channels; (7) the data was expressed in % to total number of DAPI^+^ cells.

**Polarized light microscopy.** Upon Picrosirius red staining, polarized light microscopy was performed with four representative animals/genotype at day 21 post-injury. OLYMPUS BX51 microscope (OLYMPUS, Shinjuku, Tokio, Japan) equipped with a polarized filter was used. Each tissue section was analyzed using the identical analyzer-polarizer settings allowing direct comparison between the specimens. NIKON digital camera DS-Fi2 and NIKON D3-L3 software (Nikon, Chiyoda, Tokio, Japan) were used for image acquisition. In order to cover the entire tendon area, consecutive images were taken manually and digitally stitched as mosaic with Adobe Photoshop version: 22.5.1 (Adobe System, CA, USA).

**Transmission electron microscopy (TEM).** Injured and contralateral Achilles tendons were explanted at day 21 and 100 post-injury (n=3/genotype/time point), fixed in Karnovsky (0.1 M cacodylate-buffer with 2.5% glutaraldehyde and 2% paraformaldehyde), enclosed in 4% low melting agarose, post-fixed in 1% osmium tetroxide (pH 7.3), dehydrated in graded ethanol, and embedded in the EMbed-812 epoxy resin (all reagents Science Services, Munich, Germany). After 48 h heat polymerization at 60 °C, semi-thin sections (0.8 μm) were prepared and stained with 1% toluidine blue and basic fuchsin to choose representative areas for further analysis. Ultrathin sections (0.08 μm) were collected using a Reichert Ultracut-S microtome (Leica, Bensheim, Germany), mounted on copper-grids, and stained with aqueous 2% uranyl acetate and lead citrate solutions (10 min each). The ultrathin sections for further analysis came from the injury region and from mid-portion in contralateral tendons, and were analyzed in a LEO912AB transmission electron microscope (Zeiss, Oberkochen, Germany) operating at 100 kV. Findings were documented with a side-mounted 2 k x 2 k-CCD-camera (TRS, Moorenweis, Germany). The iTEM software (Olympus, Tokyo, Japan) was used to measure collagen fibril diameter. Five images (40000x magnification)/tendon (n=2/group/time point) were used for quantification, resulting in average of 2800 fibrils/group/time point being analyzed (Supplementary Table 2).

**Micro-computed tomography (µCT).** Muscle-Achilles tendon-calcaneus complex (contralateral, non-injured and injured Achilles tendons) were excised and collected 100 days post-injury (n=4-8 animals/genotype), fixed as described above and scans were performed with the µCT system Phoenix v|tome|x s 240/180 (GE Sensing & Inspection Technologies, Frankfurt am Main, Germany). The scanning parameters for *Tnmd^-/-^* and WT hindlimbs were as follows: 50 kV voltage, 620 μA current, 500 ms time, 2000 images, voxel size 10 μm. Reconstructed volumes were processed using the manufacturer’s software phoenix datos|x 2 reconstruction 2.4.0. The threshold value defining the material and background was set to 0.12 absorption coefficient. The 3D images, the surface area and volume parameters were obtained with the software Volume Graphics VG Studio Max 2.2.3 (Volume Graphics, Heidelberg, Germany). The ROIs were defined as follows: ROI 1 (in red) start was set above the calcaneus (section plane at tibia base) and extended proximally 2.50 mm; ROI 2 (in green) began at the final plane of ROI 1 and extended into the proximal direction until the end. The calcaneal-ROI (marked in magenta color) started 0.60±0.05 mm from the end of the calcaneus and encompassed 0.60±0.05 mm region towards the ankle joint; tibiofibular-ROI (marked in blue) started 3.50±0.05 mm above the calcaneus and encompassed 1.00±0.05 mm region towards the knee joint.

**Biomechanical testing.** Mouse hindlimbs were explanted, wrapped in PBS-soaked gauze and stored at -20 °C until testing. On the testing day, hindlimbs were thawed for 30 min at RT and the 6-0 prolene suture material used for the cerclage was carefully removed. The 8-0 ethilon suture material used for end-to-end reconstruction remained in order to avoid Achilles tendon tissue damage. The viscoelastic biomechanical tests were performed with day 100 specimens (non-injured, contralateral and injured, n=8-14/genotype) using LM1 machine (TA Instruments, New Castle, USA) and custom-made clamps securing the calcaneal and myotendinous junction ends (Supplementary Fig. 6A). All tests were performed in PBS-bath at RT. The testing protocol was based on Dourte et al. and Hochstrat et al. [8, 9]. In brief, tendon length and width were measured with two digital microscope cameras (Dino-Lite Digital Microscope, Hsinchu, Taiwan) positioned in a 90° angle from each other. Prior to testing, a tendon-specific pretensioning force was applied. This force was calculated as follows: the cross-sectional area of each tendon was multiplied by a pre-defined standardized stress of 0.5 MPa resulting in values between 0.18-1.96 N. In addition, the specimens were pre-conditioned with a cyclic loading between 0.5% and 1.5% strain at 0.25Hz (10 cycles) and allowed to relax at 0% for 300 s. Next, specimens were subjected to a sinusoidal testing at different strain-levels (4, 6 and 8%) and frequencies (0.01; 0,1; 1; 5 and 10Hz) as a stress-relaxation test was performed at the start of every strain-level for 10 min. The frequency of 1 Hz was used for reporting the dynamic E-modulus. After the last strain-level, tendons were returned to the pretension force and a load-to-failure ramp was applied (Supplementary Fig. 6B). The analysis of the data was carried out with a custom-written Matlab software (MathWorks, Natick, Massachusetts, USA) protocol. The parameters cross-sectional area (mm^2^), static and dynamic E-Modulus (N/mm^2^) stiffness (N/mm) and load-to-failure (N) were evaluated. Specimens, which did not complete the entire testing protocol described above were excluded from the analyses.

**RNA isolation and reverse transcription polymerase chain reaction (qRT-PCR).** Total RNA was isolated from injured *Tnmd^-/-^* and WT Achilles tendons at day 21 and day 100 post-injury (pool of n=3 per genotype/time point) using the Qiagen RNeasy Mini kit (Qiagen, Hilden, Germany) and used for qRT-PCR. For cDNA synthesis, 1 µg total RNA and Transcriptor First-Strand cDNA Synthesis kit (Roche, Mannheim, Germany) were used following manufacturer’s instructions. Custom-designed PCR plates containing primers for tendon-related genes (BioRad Laboratories, Hercules, California, USA, Cat. Nr. 10025218) were employed. Gene expression was analyzed with the ΔΔCT method and presented as fold change of *Tnmd^-/-^* to WT. Three independent PCR experiments per genotype were carried out.

**Bioinformatics analysis.** The bioinformatics analysis started, after demultiplexing, with raw FASTQ files, which were submitted to an in-house single cell mRNA analysis pipeline 0.9.5.7. The raw sequence data from six *ScxGFP^+^* cells/genotype (n=6) were trimmed with BBDuk 38.84 [10], removing remaining adapter sequences and poor-quality bases at the end of each read. Read decontamination was performed using BioBloom Tolls 2.0.13 [11] with filters for the genomes of *Mus musculus* (mm38), *Homo sapiens* (hg38), *Escherichia coli* (BL21), *Mycoplasma pneumonia* (M129), *Sphingobium sp.* (SYK-6), *Bradyrhizobium japonicum* (USDA 110), *Pichia pastoris* (GS115), *Malassezia globosa* (CBS 7966), *Aspergillus fumigatus* (AF293), and a set of viral genomes (RefSeq, 5k+ genomes). All reads that did not map exclusively to mm38 (Ensembl version 96, GRCm38 DNA primary assembly) were defined as likely contaminations and discarded from downstream processing. Sequence quality per sample was evaluated before as well as after trimming and decontamination using FastQC 0.11.9 [12] and, in addition, all samples were analyzed as a collective with MultiQC 1.9 [13]. Next, the cleaned sample reads were aligned to the reference genome mm38 with STAR 2.5.1b [14]. Reads mapping uniquely in exonic regions were counted per gene and per sample using feature counts from Subread 2.0.0 [15]. Further quality characteristics such as library complexity, using Preseq 2.0.3 [16], and the genomic origin of the reads and the 5’-3’-bias, both with QualiMap 2.2.2d [17], were assessed. The final counts table of the 12 samples (6 cells/genotype) were used further for differential gene expression analysis. The top 500 most variable genes were clustered using principal component analysis (PCA) and t-distributed stochastic neighbor embedding (t-SNE) with the Bioconductor package scater 1.14.6 [18]. Raw counts were normalized and scaled into logCPM values before proceeding to batch correction. The bias introduced by degradation were corrected using cells with better quality as reference by Combat [19] function in the Bioconductor package sva 3.34.0 [20]. Differential expression analysis was performed between *Tnmd^-/-^ ScxGFP^+^* and WT *ScxGFP^+^* cells with the Bioconductor package edgeR 3.28.1 [21] and DESeq2 1.26.0 [22]. Functional classification of differentially expressed genes (DEGs) into Gene Ontology (GO) [23] and Kyoto Encyclopedia of Genes and Genomes (KEGG) [24] categories were performed in R programming language 4.0.2 [25] using Bioconductor 1.30.10 packages. GO enrichment annotates DEGs into three categories including Biological Process (BP), Molecular Function (MF) and Cellular Component (CC) whereas KEGG annotates DEGs into pathways. Intersection genes identified as differentially expressed in both DESeq2 and EdgeR were processed for enrichment analysis and log Fold change (logFc), p-value and adjusted p-value for the intersection genes were extracted from the DESeq2 method. Furthermore, this final list of 1430 DEGs were filtered for adjusted p-value <0.05 and |logFc|> 1. GOseq 1.42.0 [26] with Wallenius noncentral hypergeometric distribution method used for GO enrichment analysis. This method uses a Probability Weighting Function (PWF) to correct for bias in gene length before performing an enrichment on DEGs. ClusterProfiler 3.18.1 [27] was used to perform Gene Set Enrichment analysis (GSEA) on KEGG terms and org.Mm.eg.db 312.0 was imported to map the gene symbols to Entrez gene identifiers in the mice database. For Gene Set Enrichment Analysis (GSEA), filtered genes were ranked according to their log Fold Change values. All figures were plotted using ggplot2 3.3.3 [28]. The scRNA-Seq datasets used for the analysis have been deposited into the Gene Expression Omnibus database at the National Center for Biotechnology Information under the GEO accession number GSE179454 (https://www.ncbi.nlm.nih.gov/geo/query/acc.cgi?acc =GSE179454).

**References**

1. Stoll C, John T, Conrad C, Lohan A, Hondke S, Ertel W et al. Healing parameters in a rabbit partial tendon defect following tenocyte/biomaterial implantation. Biomaterials 2011; 32(21):4806–15.

2. Lin D, Alberton P, Caceres MD, Volkmer E, Schieker M, Docheva D. Tenomodulin is essential for prevention of adipocyte accumulation and fibrovascular scar formation during early tendon healing. Cell Death Dis 2017; 8(10):e3116.

3. Docheva D, Hunziker EB, Fässler R, Brandau O. Tenomodulin is necessary for tenocyte proliferation and tendon maturation. Mol Cell Biol 2005; 25(2):699–705.

4. Pryce BA, Brent AE, Murchison ND, Tabin CJ, Schweitzer R. Generation of transgenic tendon reporters, ScxGFP and ScxAP, using regulatory elements of the scleraxis gene. Dev Dyn 2007; 236(6):1677–82.

5. Somerville JM, Aspden RM, Armour KE, Armour KJ, Reid DM. Growth of C57BL/6 mice and the material and mechanical properties of cortical bone from the tibia. Calcif Tissue Int 2004; 74(5):469–75.

6. Lemme NJ, Li NY, DeFroda SF, Kleiner J, Owens BD. Epidemiology of Achilles Tendon Ruptures in the United States: Athletic and Nonathletic Injuries From 2012 to 2016. Orthop J Sports Med 2018; 6(11):2325967118808238.

7. Lillie RD, Tracy RE, Pizzolato P, Donaldson PT, Reynolds C. Differential staining of collagen types in paraffin sections: a color change in degraded forms. Virchows Arch A Pathol Anat Histol 1980; 386(2):153–9.

8. Dourte LM, Pathmanathan L, Jawad AF, Iozzo RV, Mienaltowski MJ, Birk DE et al. Influence of decorin on the mechanical, compositional, and structural properties of the mouse patellar tendon. J Biomech Eng 2012; 134(3):31005.

9. Hochstrat E, Müller M, Frank A, Michel P, Hansen U, Raschke MJ et al. Cryopreservation of tendon tissue using dimethyl sulfoxide combines conserved cell vitality with maintained biomechanical features. PLoS One 2019; 14(4):e0215595.

10. JGI DataScience. BBtools software suite 2019. Available from: URL: https://jgi.doe.gov/data-and-tools/bbtools.

11. Chu J, Sadeghi S, Raymond A, Jackman SD, Nip KM, Mar R et al. BioBloom tools: fast, accurate and memory-efficient host species sequence screening using bloom filters. Bioinformatics 2014; 30(23):3402–4.

12. Babraham Bioinformatics 2019. FastQC. Available from: URL: https://www.bioinformatics.babraham.ac.uk/projects/fastqc.

13. Ewels P, Magnusson M, Lundin S, Käller M. MultiQC: summarize analysis results for multiple tools and samples in a single report. Bioinformatics 2016; 32(19):3047–8.

14. Dobin A, Davis CA, Schlesinger F, Drenkow J, Zaleski C, Jha S et al. STAR: ultrafast universal RNA-seq aligner. Bioinformatics 2013; 29(1):15–21.

15. Liao Y, Smyth GK, Shi W. The Subread aligner: fast, accurate and scalable read mapping by seed-and-vote. Nucleic Acids Res 2013; 41(10):e108.

16. Deng C, Daley T, Smith AD. Applications of species accumulation curves in large-scale biological data analysis. Quant Biol 2015; 3(3):135–44.

17. Okonechnikov K, Conesa A, García-Alcalde F. Qualimap 2: advanced multi-sample quality control for high-throughput sequencing data. Bioinformatics 2016; 32(2):292–4.

18. McCarthy DJ, Campbell KR, Lun ATL, Wills QF. scater: pre-processing, quality control, normalisation and visualisation of single-cell RNA-seq data in R; 2016.

19. Leek JT, Johnson WE, Parker HS, Jaffe AE, Storey JD. The sva package for removing batch effects and other unwanted variation in high-throughput experiments. Bioinformatics 2012; 28(6):882–3.

20. Johnson WE, Li C, Rabinovic A. Adjusting batch effects in microarray expression data using empirical Bayes methods. Biostatistics 2007; 8(1):118–27.

21. Robinson MD, McCarthy DJ, Smyth GK. edgeR: a Bioconductor package for differential expression analysis of digital gene expression data. Bioinformatics 2010; 26(1):139–40.

22. Love MI, Huber W, Anders S. Moderated estimation of fold change and dispersion for RNA-seq data with DESeq2. Genome Biol 2014; 15(12):550.

23. Ashburner M, Ball CA, Blake JA, Botstein D, Butler H, Cherry JM et al. Gene ontology: tool for the unification of biology. The Gene Ontology Consortium. Nat Genet 2000; 25(1):25–9.

24. Kanehisa M, Goto S, Furumichi M, Tanabe M, Hirakawa M. KEGG for representation and analysis of molecular networks involving diseases and drugs. Nucleic Acids Res 2010; 38(Database issue):D355-60.

25. R Core Team 2021. R: A Language and Environment for Statistical Computing. R Foundation for Statistical Computing. Vienna, Austria. Available from: URL: www.r-project.org/.

26. Young MD, Wakefield MJ, Smyth GK, Oshlack A. Gene ontology analysis for RNA-seq: accounting for selection bias. Genome Biol 2010; 11(2):R14.

27. Yu G, Wang L-G, Han Y, He Q-Y. clusterProfiler: an R package for comparing biological themes among gene clusters. OMICS 2012; 16(5):284–7.

28. Hadley Wickham. ggplot2: Elegant Graphics for Data Analysis. Springer-Verlag New York; 2016. Available from: URL: https://ggplot2.tidyverse.org.
