# Supplementary figures and images for: Tenomodulin knockout mice exhibit worse late healing outcomes with augmented trauma-induced heterotopic ossification of Achilles tendon
Source: Cell Death Dis. 2021 Nov 5;12(11):1049. doi: 10.1038/s41419-021-04298-z (PMC8571417; doi:10.1038/s41419-021-04298-z)

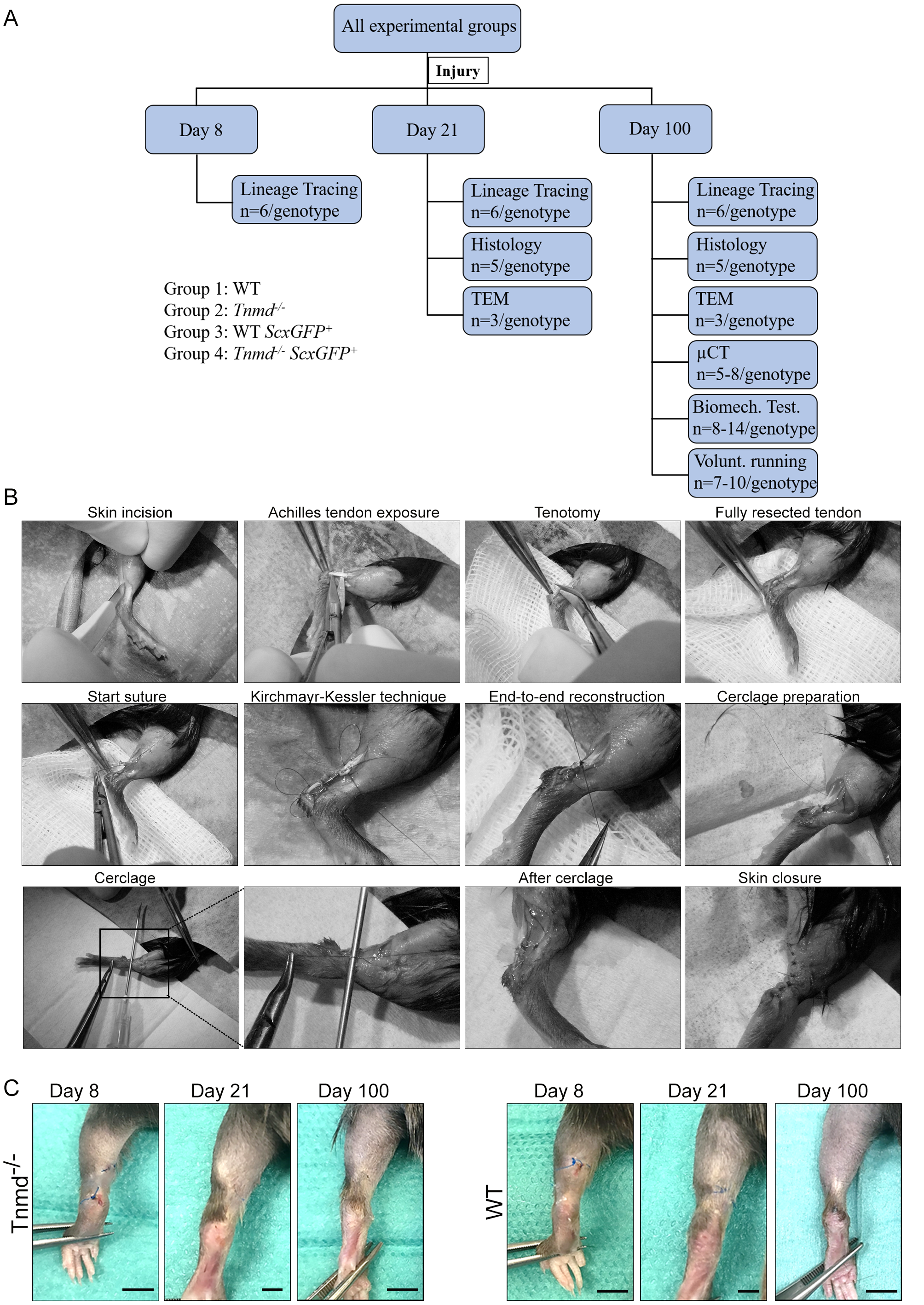

Supplement: Supplementary file 2 — Supplementary Fig. 1 [file 41419_2021_4298_MOESM2_ESM.tif]

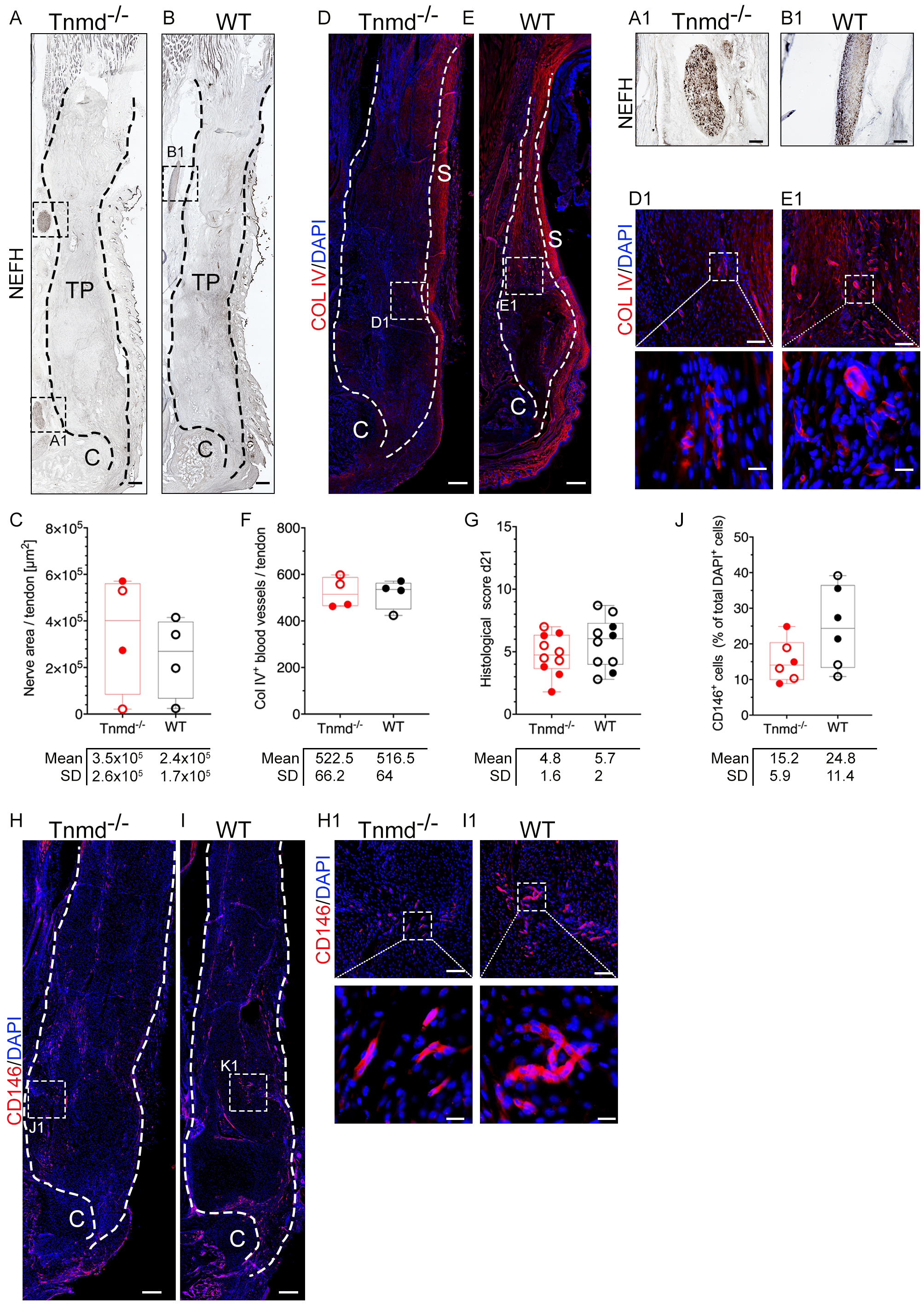

Supplement: Supplementary file 3 — Supplementary Fig. 2 [file 41419_2021_4298_MOESM3_ESM.tif]

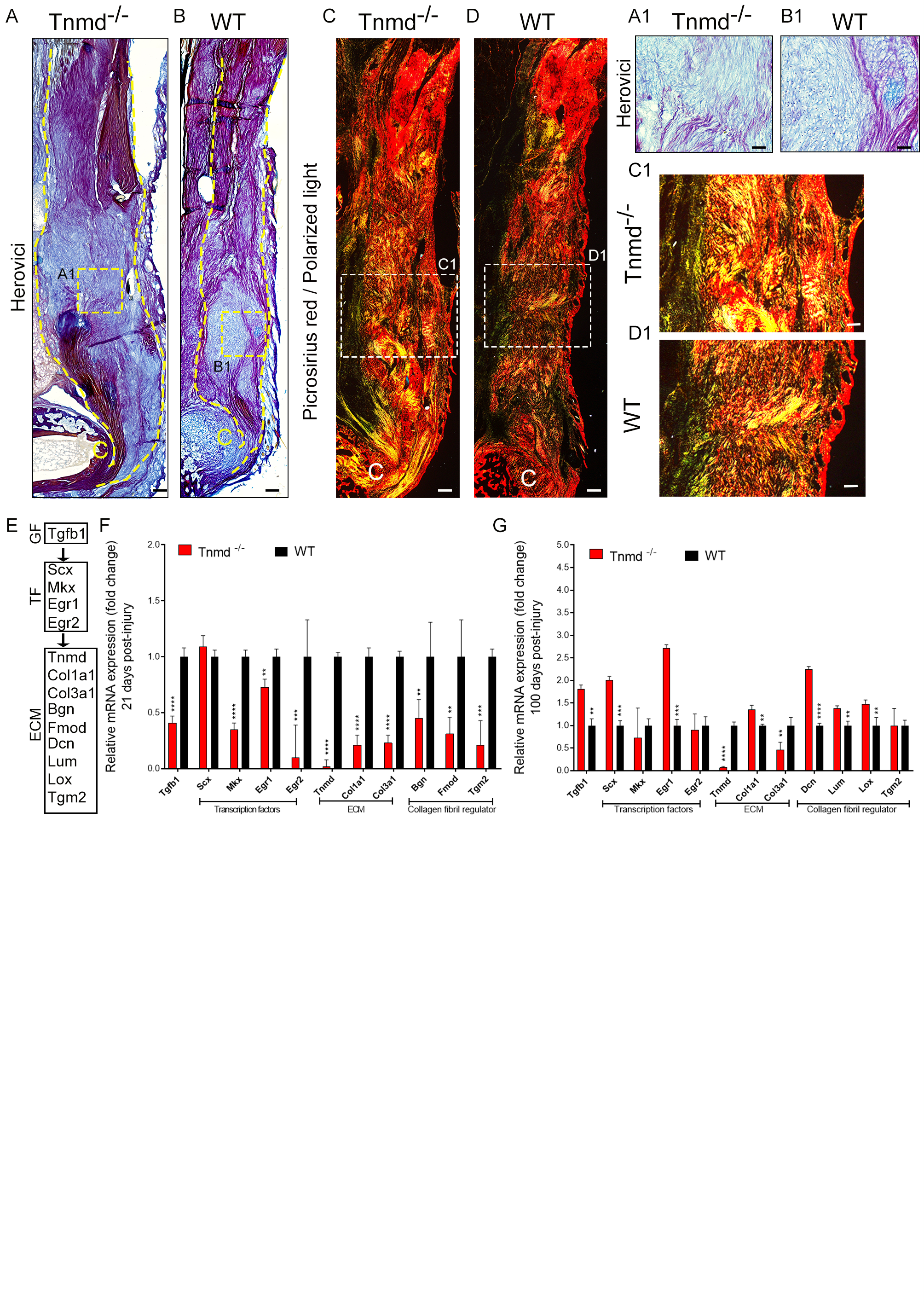

Supplement: Supplementary file 4 — Supplementary Fig. 3 [file 41419_2021_4298_MOESM4_ESM.tif]

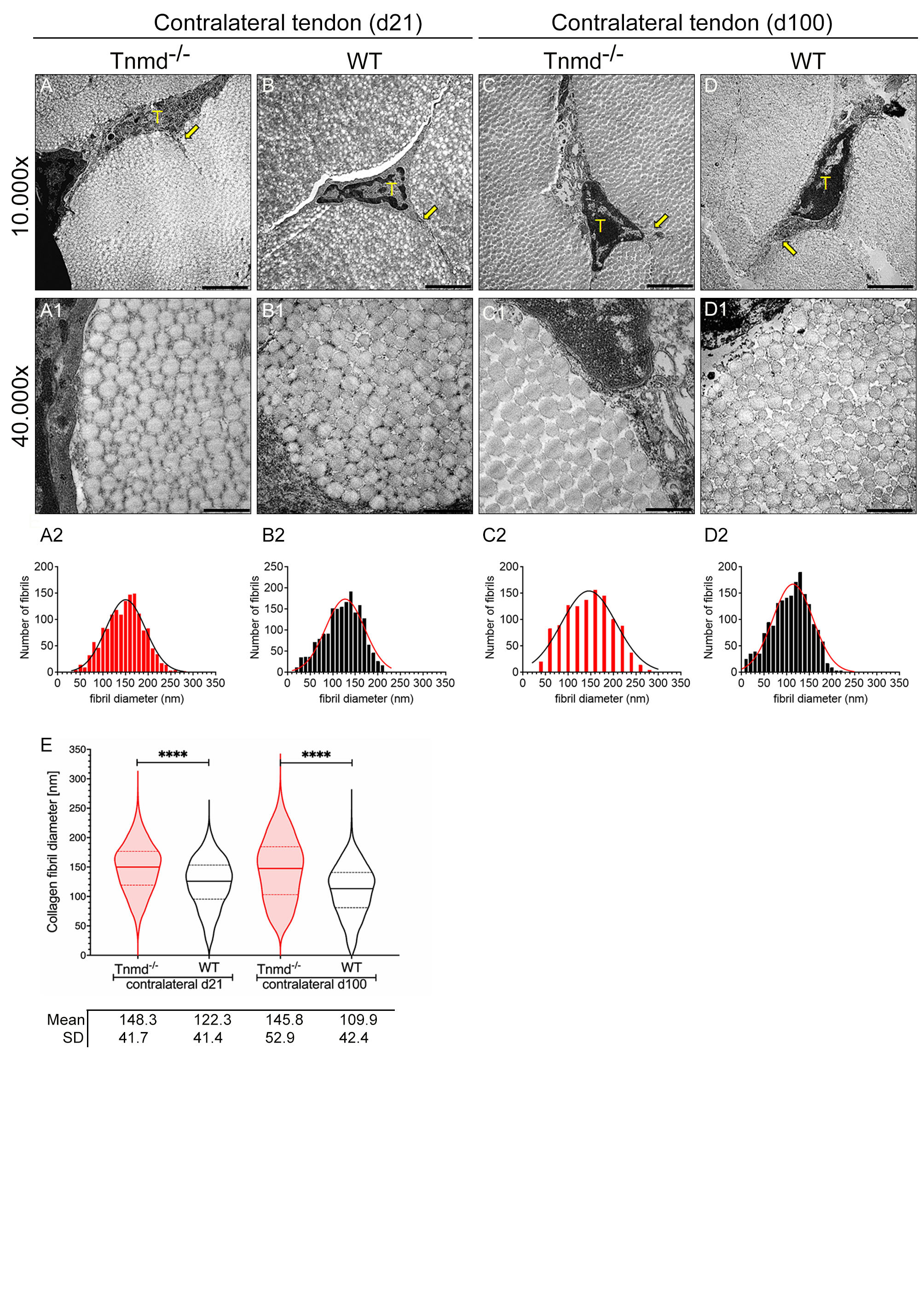

Supplement: Supplementary file 5 — Supplementary Fig. 4 [file 41419_2021_4298_MOESM5_ESM.tif]

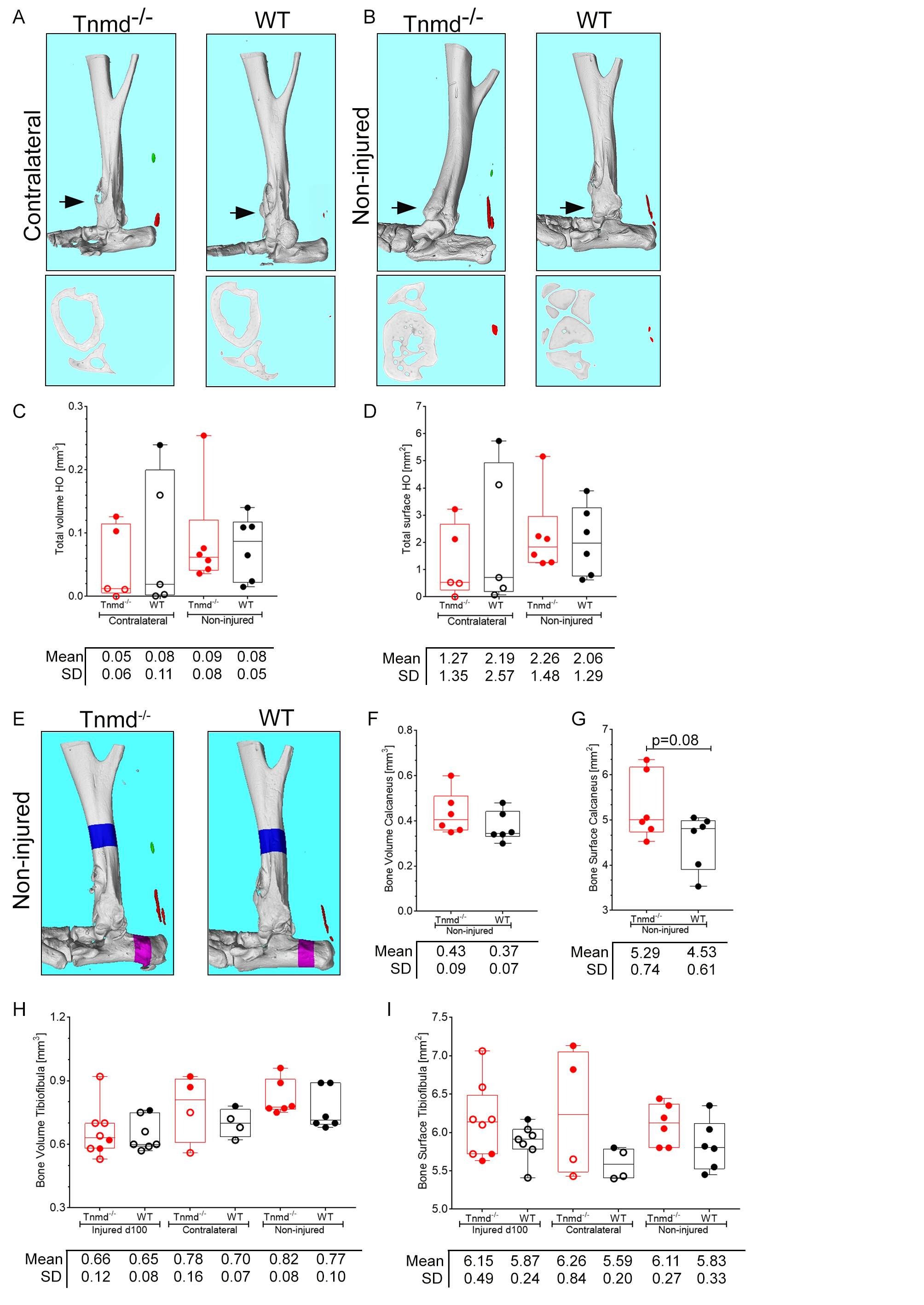

Supplement: Supplementary file 6 — Supplementary Fig. 5 [file 41419_2021_4298_MOESM6_ESM.tif]

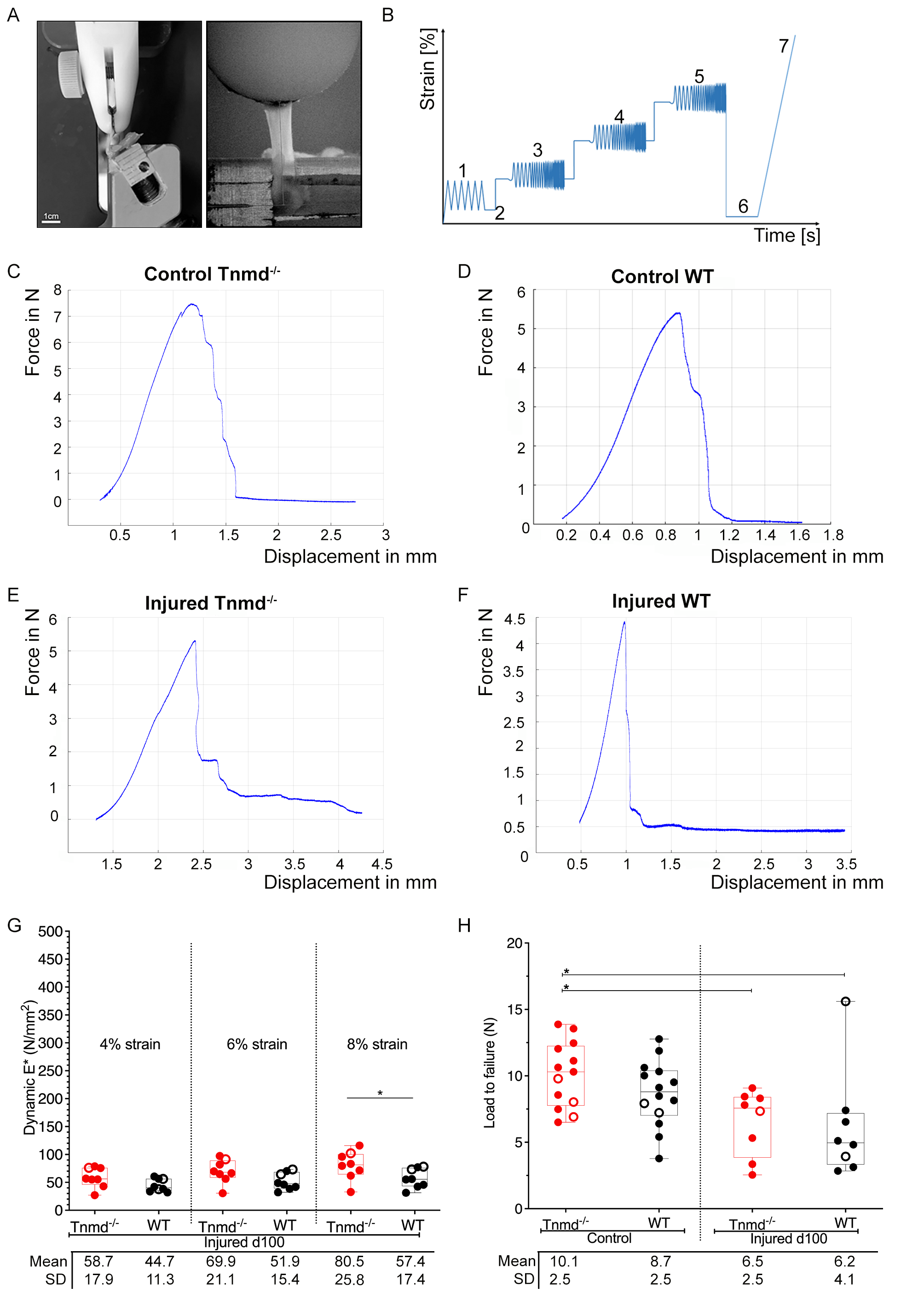

Supplement: Supplementary file 7 — Supplementary Fig. 6 [file 41419_2021_4298_MOESM7_ESM.tif]
